# The effect of universal testing and treatment for HIV on health-related quality of life – An analysis of data from the HPTN 071 (PopART) cluster randomised trial

**DOI:** 10.1016/j.ssmph.2023.101473

**Published:** 2023-07-22

**Authors:** Katherine Davis, Michael Pickles, Simon Gregson, James R. Hargreaves, Helen Ayles, Peter Bock, Triantafyllos Pliakas, Ranjeeta Thomas, Julius Ohrnberger, Justin Bwalya, Nomtha Bell-Mandla, Kwame Shanaube, William Probert, Graeme Hoddinott, Virginia Bond, Richard Hayes, Sarah Fidler, Katharina Hauck, the HPTN 071 (PopART) Study Team

**Affiliations:** a MRC Centre for Global Infectious Disease Analysis, School of Public Health, Imperial College London, London, W2 1PG, UK; b Abdul Latif Jameel Institute for Disease and Emergency Analytics, School of Public Health, Imperial College London, London, W2 1PG, UK; c Department of Public Health, Environments and Society, Faculty of Public Health and Policy, London School of Hygiene and Tropical Medicine, London, WC1E 7HT, UK; d Department of Clinical Research, Faculty of Infectious and Tropical Diseases, London School of Hygiene and Tropical Medicine, London, WC1E 7HT, UK; e Zambart, School of Medicine, University of Zambia, Lusaka, Zambia; f Desmond Tutu TB Centre, Department of Paediatrics and Child Health, Faculty of Medicine and Health, University of Stellenbosch, Cape Town, South Africa; g Department of Health Policy, London School of Economics, London, WC2A 2AE, UK; h Big Data Institute, Nuffield Department of Medicine, University of Oxford, Oxford, OX3 7LF, UK; i Department of Global Health and Development, Faculty of Public Health and Policy, London School of Hygiene and Tropical Medicine, London, WC1E 7HT, UK; j Department of Infectious Disease Epidemiology, Faculty of Epidemiology and Population Health, London School of Hygiene and Tropical Medicine, London, WC1E 7HT, UK; k Department of Infectious Disease, Faculty of Medicine, Imperial College London, W2 1PG, UK

**Keywords:** HIV, Universal testing and treatment, Health-related quality of life, Zambia, South Africa, Cluster randomised controlled trial

## Abstract

**Background:**

HIV treatment has clear Health-Related Quality-of-Life (HRQoL) benefits. However, little is known about how Universal Testing and Treatment (UTT) for HIV affects HRQoL. This study aimed to examine the effect of a combination prevention intervention, including UTT, on HRQoL among People Living with HIV (PLHIV).

**Methods:**

Data were from HPTN 071 (PopART), a three-arm cluster randomised controlled trial in 21 communities in Zambia and South Africa (2013–2018). Arm A received the full UTT intervention of door-to-door HIV testing plus access to antiretroviral therapy (ART) regardless of CD4 count, Arm B received the intervention but followed national treatment guidelines (universal ART from 2016), and Arm C received standard care. The intervention effect was measured in a cohort of randomly selected adults, over 36 months. HRQoL scores, and the prevalence of problems in five HRQoL dimensions (mobility, self-care, performing daily activities, pain/discomfort, anxiety/depression) were assessed among all participants using the EuroQol-5-dimensions-5-levels questionnaire (EQ-5D-5L). We compared HRQoL among PLHIV with laboratory confirmed HIV status between arms, using adjusted two-stage cluster-level analyses.

**Results:**

At baseline, 7,856 PLHIV provided HRQoL data. At 36 months, the mean HRQoL score was 0.892 (95% confidence interval: 0.887–0.898) in Arm A, 0.886 (0.877–0.894) in Arm B and 0.888 (0.884–0.892) in Arm C. There was no evidence of a difference in HRQoL scores between arms (A vs C, adjusted mean difference: 0.003, -0.001-0.006; B vs C: -0.004, -0.014-0.005). The prevalence of problems with pain/discomfort was lower in Arm A than C (adjusted prevalence ratio: 0.37, 0.14–0.97). There was no evidence of differences for other HRQoL dimensions.

**Conclusions:**

The intervention did not change overall HRQoL, suggesting that raising HRQoL among PLHIV might require more than improved testing and treatment. However, PLHIV had fewer problems with pain/discomfort under the full intervention; this benefit of UTT should be maximised during roll-out.

## Highlights

• At baseline of the trial, overall health-related quality of life (HRQoL) was high.
• After 3 years, the intervention was not associated with greater overall HRQoL.
• At baseline, pain/discomfort was the aspect of HRQoL with most problems reported.
• At endpoint, problems with pain/discomfort were rarer under the full intervention.
• Raising overall HRQoL needs more than improved access to testing and treatment.

## Abbreviations

aMD: adjusted mean difference
aPR: adjusted prevalence ratio
ART: antiretroviral therapy
CI: confidence interval
EQ-5D-5L: EuroQol five dimensions, five levels questionnaire
HRQoL: health-related quality of life
PC: population cohort
PLHIV: people living with HIV
UTT: universal testing and treatment

## 1 Introduction

In 2021, approximately 38 million people were living with HIV globally (UNAIDS, 2022a). HIV-related disease continues to be a leading cause of death in the regions that are worst affected by HIV, but survival of People Living with HIV (PLHIV) has improved (UNAIDS, 2022b).

As life expectancy of PLHIV has increased, interest in enhancing their Health-Related Quality of Life (HRQoL) has grown (Safreed-Harmon et al., 2019). An effective way to increase HRQoL among PLHIV might be Universal Testing and Treatment (UTT) (Orne-Gliemann et al., 2015). UTT involves delivering HIV testing to everyone in an area, with antiretroviral therapy (ART) initiation for anyone with a positive diagnosis (Hayes et al., 2014). UTT is a population-based strategy that can be used in regions with high HIV prevalence, such as eastern and southern Africa (Hayes et al., 2011; Perriat et al., 2018). Research into UTT has increased over the last decade, after several modelling studies indicated that UTT could reduce the incidence of HIV and the burden of disease among PLHIV (Granich et al., 2009; Havlir et al., 2020; Hayes et al., 2011; Hontelez et al., 2013). By diagnosing people earlier and initiating them on ART at higher CD4 counts than under standard care, UTT reduces morbidity among PLHIV and may improve HRQoL (Bunupuradah et al., 2013; Kumar et al., 2021; Lifson et al., 2017; The INSIGHT START Study Group, 2015).

It is possible, however, that gains in HRQoL from UTT may be at least partially offset by short-run unintended negative impacts (Brazier et al., 2019; Havlir et al., 2020; Orne-Gliemann et al., 2015). If these negative impacts are severe, they may need to be addressed through additional interventions provided alongside UTT. An example of a negative impact is the increased symptoms of anxiety and depression that PLHIV who test earlier than they would have under standard-of-care may suffer in the short- or medium-term (Kagee et al., 2017; Ramirez-Avila et al., 2012). Similarly, side effects of ART among PLHIV in good health, who would not otherwise be receiving care, may also decrease HRQoL (Renju et al., 2017). The existence of both plausible negative short-term impacts of UTT and plausible positive short-term impacts of UTT, leads to uncertainty about the overall measured impact of UTT on HRQoL, which may bias morbidity impact estimates from economic evaluations and affect policy decisions (Whitehead & Ali, 2010).

Despite the uncertainty about the effect of UTT on HRQoL, there is limited research comparing the HRQoL of PLHIV who were exposed to UTT to the HRQoL of PLHIV who were not exposed. Three randomised trials have measured the impact of providing only universal (or early) treatment on HRQoL among PLHIV in care and all found that universal treatment was associated with improvements in at least some HRQoL dimensions (Bunupuradah et al., 2013; Kumar et al., 2021; Lifson et al., 2017). However, insights into the combined effect of universal testing and universal treatment on HRQoL are rare. Moreover, the previous studies of universal treatment all excluded PLHIV who were not initiating treatment, PLHIV who had previously taken treatment, or PLHIV who had advanced disease, which limits understanding of the effects of UTT on HRQoL across the population of PLHIV (Bunupuradah et al., 2013; Kumar et al., 2021; Lifson et al., 2017). Essentially, the samples of PLHIV in previous studies were often not representative samples of the underlying populations of PLHIV. This prevents policymakers from judging the population-level impact of UTT on HRQoL.

To help close the evidence gap, this study examined how UTT affects HRQoL among PLHIV using data from the HPTN 071 (PopART) trial (Hayes et al., 2014). HPTN 071 (PopART) was a cluster randomised controlled trial, which assessed the impact of a combination prevention intervention, including UTT, on HIV incidence in Zambia and South Africa. During the trial, universal treatment was integrated into national HIV guidelines, and in future, universal testing in communities with high HIV prevalence may be integrated too, further highlighting the urgent need to fully understand the effects of UTT and ameliorate any negative consequences (Hayes et al., 2014, 2019). In the current study, the effects of the intervention on aggregate HRQoL and on five constituent HRQoL dimensions were explored.

## 2 Material and methods

### 2.1 Trial structure

#### 2.1.1 Trial design

The design of HPTN 071 (PopART) is described in detail in the appendix (section 1) and trial protocol (https://www.hptn.org/research/studies/hptn071#views-field-field-public-files). Briefly, the study was a matched three-arm cluster randomised controlled trial, conducted in 21 urban and peri-urban communities in South Africa and Zambia between 2013 and 2018 (Fig. A.1) (Hayes et al., 2019).

In the trial, Arm A communities (n = 7) received the full combination prevention intervention. This included door-to-door HIV testing, support with linkage to HIV care and ART adherence, and access to ART regardless of CD4 count. Testing and referral for treatment was carried out by a cadre of community health workers. Arm B communities (n = 7) received the same intervention, including door-to-door HIV testing, but with ART provided according to national guidelines. Arm C communities (n = 7) received standard-of-care, with HIV testing and ART offered according to national guidelines (Hayes et al., 2014). During 2016, national guidelines switched from providing ART based on CD4 counts to universal provision irrespective of CD4 counts (Fig. A.2) (Hayes et al., 2019).

#### 2.1.2 Outcome evaluation

A Population Cohort (PC) was used to assess the intervention (Hayes et al., 2019). At baseline, the PC was populated by random sampling of adults aged 18–44 years. PC participants were then surveyed at baseline, 12, 24 and 36 months. Additional enrolment occurred at 12 and 24 months where recruitment targets were not met (Hayes et al., 2019).

At each survey round, information was gathered on socio-demographics, HRQoL, and use of HIV services. Blood samples were also taken for HIV testing (Hayes et al., 2019). To monitor HRQoL, PLHIV and HIV-negative participants were invited to answer the EuroQol five dimensions, five levels questionnaire (EQ-5D-5L). The EQ-5D-5L measures HRQoL in five dimensions (mobility, self-care, performance of daily activities, pain/discomfort, and anxiety/depression). Each dimension is measured through a single question that assesses the extent of problems that someone experienced in the dimension on day of the survey, over five levels from ‘no problems’ to ‘unable to/extreme problems’ (EuroQol Research Foundation, 2019). The EQ-5D-5L has been used previously among PLHIV in diverse settings and has well established reliability and validity (Miners et al., 2014; Thomas et al., 2017; Tran et al., 2012; Wu et al., 2013).

### 2.2 Statistical methods

#### 2.2.1 Study sample

A cohort of PC participants who were present at baseline (to allow adjustment for baseline imbalances), who were found to be HIV-positive during testing at baseline, and who had completed the EQ-5D-5L during at least one survey was selected for analysis (Fig. A.3).

#### 2.2.2 Outcome measures

To assess overall HRQoL, responses across the five EQ-5D-5L questions were characterised with a single score using the Zimbabwean mapping of responses to scores, because, although a South African mapping is available, a Zambian mapping is not (Van Hout et al., 2012). Scores were analysed on a scale from 0.001 (worst) to 0.9 (best), as described in the appendix (section 1). For analyses of HRQoL dimensions, responses were collapsed to binary variables that captured whether participants reported any problems (EuroQol Research Foundation, 2019).

#### 2.2.3 Unadjusted analyses of the effect of the intervention on HRQoL scores

The effect of the intervention was examined by comparing aggregate HRQoL scores and the prevalence of problems in each dimension of HRQoL between trial arms. Initially, an assessment of balance between the arms at baseline was performed. Once this was complete, the unadjusted effect of the intervention on HRQoL score at 36 months was explored. Similarities between interventions in Arms A and B after changes to ART guidelines allowed comparisons of pooled data from Arms A and B (Arms A + B) with Arm C, as well as comparisons of Arm A with Arm C and Arm B with Arm C. For each comparison, the difference in mean HRQoL scores for every matched pair of communities was computed and the mean of the differences was calculated. Using the t distribution, 95% Confidence Intervals (CIs) for the mean difference were generated and a paired *t*-test on the community means was employed to assess evidence for a difference between arms.

#### 2.2.4 Adjusted analyses of the effect of the intervention on HRQoL scores

To perform adjusted analyses for each comparison, a two-stage approach, which is recommended for cluster randomised controlled trials with fewer than 15 clusters per arm, was used (Hayes & Moulton, 2017). In the first stage, a beta regression model was run with individual-level data, assuming that there was no intervention effect, with HRQoL score as the outcome. Beta regression was chosen as it is more robust than other commonly used approaches for modelling HRQoL (Basu & Manca, 2012; Thomas et al., 2017). Covariates included age, sex, and the interaction between age and sex, which were treated as *a priori* confounders, and triplet, reflecting the matched study design. Additional covariates that were added because they were unbalanced at baseline among PLHIV included language used in the questionnaire (five languages in Zambia, three in South Africa), which may affect interpretation of the EQ-5D-5L, and, baseline wealth, baseline position in the HIV care cascade (unaware of status, aware of status but not in care, in care but not on ART, used ART less than five years, used ART for five or more years) and baseline HRQoL scores. Fitted HRQoL scores were extracted from the beta regression model and used to produce the expected mean HRQoL score for each community in the absence of intervention. For each community, the expected HRQoL scores were then subtracted from observed scores to give a difference residual. In the second stage, the mean difference in residuals between communities in the two arms was calculated to give an adjusted effect estimate (adjusted Mean Difference, aMD), with the t distribution used for calculation of 95% CIs and a paired *t*-test used to compare the residuals between intervention and control arms.

#### 2.2.5 Unadjusted and adjusted analyses of the effect of the intervention on dimensions of HRQoL

A similar approach was used for analyses that explored the effect of the intervention on the prevalence of problems in each HRQoL dimension (Van Hout et al., 2012). First, prevalence estimates were determined for each community. Then, to calculate an unadjusted effect estimate, logs of the prevalence in each community were taken and the mean difference in the log prevalences between arms was calculated. Paired t-tests were used to assess the evidence for a difference in the log prevalences and the t distribution was used to estimate 95% CIs on the log scale. Prevalence ratios and 95% CIs were then determined by exponentiation. For the adjusted analysis, the two-stage approach was as described above, but logistic regression replaced beta regression, baseline prevalence of problems was adjusted for rather than HRQoL scores, and the observed and expected data were compared differently. Specifically, the observed prevalence for each community was divided by the expected prevalence to give a ratio residual and logs of the ratio residuals were taken. The log ratio residuals were compared between arms in the same manner as the unadjusted log prevalences, with exponentiation used to estimate the adjusted Prevalence Ratio (aPR) and 95% CIs. In some analyses, there were communities with no individuals reporting problems. To avoid taking a log of zero, small constants proportional to the number of PLHIV were added to the numerator and denominator of the prevalence (Hayes & Moulton, 2017).

#### 2.2.6 Subgroup and exploratory analyses

Subgroup analyses stratified by sex were performed, following the approach described above. For some HRQoL dimensions, few men reported having problems, and so estimates were not calculated. Outcomes at 12 and 24 months were also examined using the same approaches.

As exploratory analyses, we assessed the effect of including people who seroconverted during the trial in the study sample and performed subgroup analyses stratified by ART usage at endpoint (appendix, section 2).

### 2.3 Ethics

Ethical approval for the trial was provided by institutional review boards at the London School of Hygiene and Tropical Medicine, Stellenbosch University, and the University of Zambia. Participants provided written informed consent.

### 2.4 Role of the funding source

The funders of the study had no role in the study design, collection, analysis, and interpretation of data, writing of the article, or decision to submit for publication.

### 2.5 Patient and public involvement

The public have been involved through-out the HPTN 071 (PopART) research process. Before the trial began, individuals from existing representative structures, including members of community advisory boards from previous studies in the area, local opinion leaders, and government stakeholders, were consulted about study design. A broad-brush survey approach was also used to provide a rapid assessment of the HIV prevention, treatment, and care landscapes prior to trial initiation. During the trial, multiple involvement and engagement mechanisms were employed to understand and improve the trial, including meetings with community advisory boards for adults and adolescents, and connections with civil society groups. Links with the public were also drawn upon at the end of intervention delivery to understand how results should be disseminated, with the first dissemination round using a community dialogue approach, which focused on what results meant to the communities. Further dissemination of study results, such as those presented here, will continue to involve communities in decision making.

## 3 Results

### 3.1 Enrolment and follow-up

**Fig. 1:**
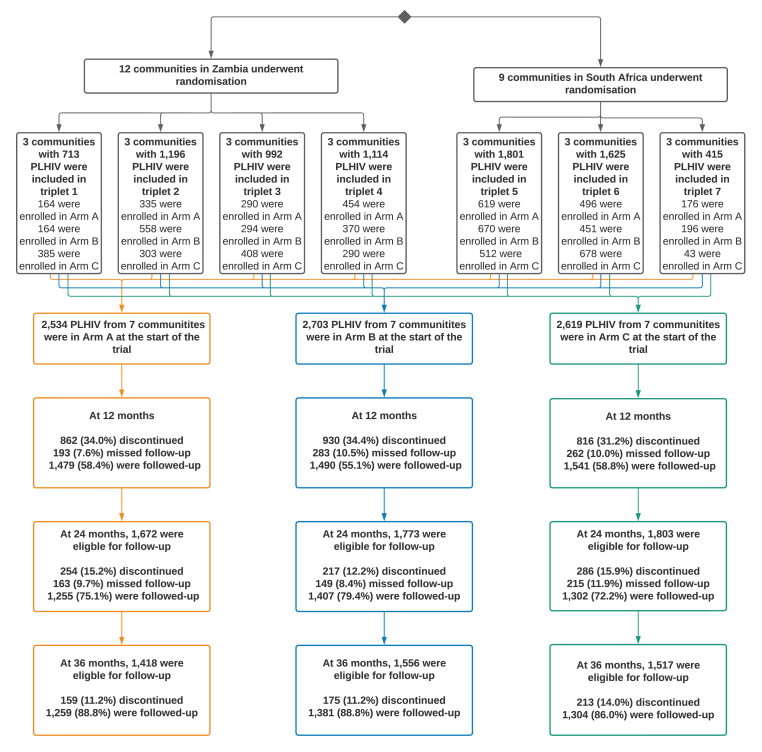
Enrolment and follow-up of the cohort of People Living with HIV (PLHIV) The numbers of participants enrolled in each triplet are shown in the black boxes. The coloured boxes track those who provided health-related quality of life information across the surveys. Participants who discontinued either left the Population Cohort or stopped answering the questions about health-related quality of life. Participants who missed follow-up either missed one survey, but then completed a later survey, or did not answer the questions on health-related quality of life during one survey, but then chose to answer at a later survey.

At baseline, 7,856 PLHIV were enrolled and provided HRQoL data. Discontinuation rates were similar between study arms (Fig. 1).

**Fig. 2:**
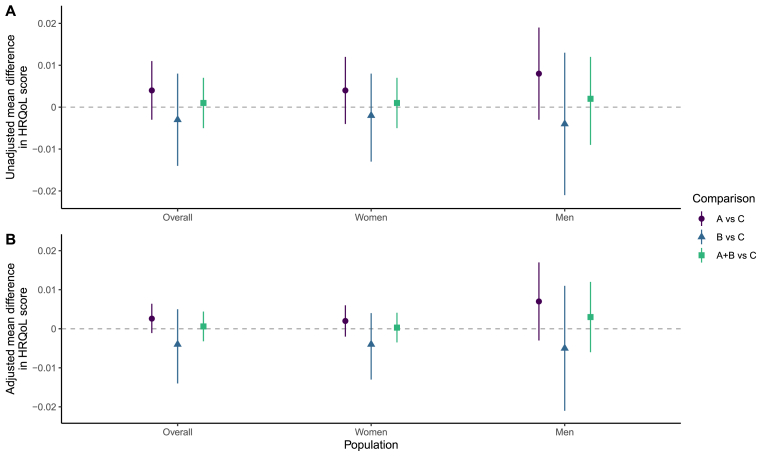
Effect of universal testing and treatment on Health-Related Quality of Life (HRQoL) score at 36 months A) Unadjusted estimates of the mean difference in HRQoL score between the arms. B) Adjusted estimates of the mean difference in HRQoL score between the arms. Overall mean differences were adjusted for age, sex, language(s) used, baseline wealth, baseline position in the HIV care cascade and baseline HRQoL score. Analyses stratified by sex were adjusted for the same variables, except sex was excluded. Error bars: 95% confidence intervals.

**Fig. 3:**
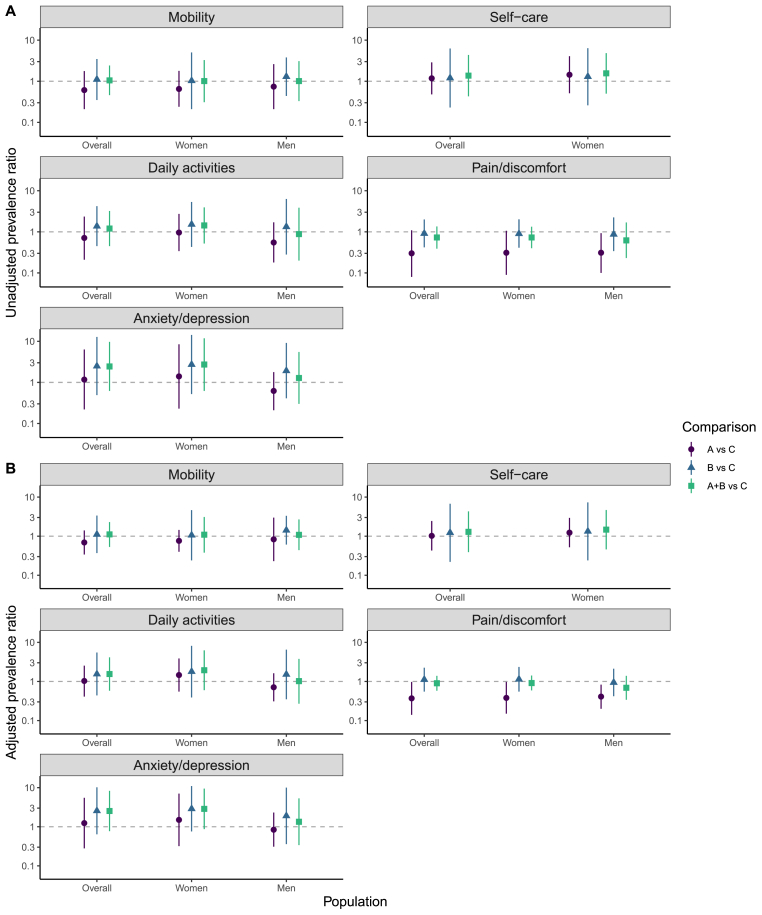
Effect of universal testing and treatment on dimensions of Health-Related Quality of Life (HRQoL) at 36 months (A) Unadjusted estimates of prevalence ratios for problems with each of the five HRQoL dimensions. B) Adjusted estimates of prevalence ratios for problems with each of the five HRQoL dimensions. Overall prevalence ratios were adjusted for age, sex, language(s) used, baseline wealth, baseline position in the HIV care cascade and baseline prevalence of problems in each HRQoL dimension. Analyses stratified by sex were adjusted for the same variables, except sex was excluded. Prevalence ratios less than one indicate fewer problems under the intervention and a log scale is used. The very small number of men reporting problems with self-care means that estimates are not reported for this outcome. Error bars: 95% confidence intervals.

### 3.2 Baseline comparisons

There were more women (84.4%) than men in the cohort and participants had a median age of 31 (Table 1). Nearly half of participants self-reported that they were HIV-positive (48.8%). Of these, approximately two-thirds reported using ART (68.4%).

**Table 1:** Characteristics of the cohort of people living with HIV at baseline.

	Overall, N = 7,856^a^	Arm A, N = 2,534^a^	Arm B, N = 2,703^a^	Arm C, N = 2,619^a^
Sex				
Female	6,631 (84.4%)	2,131 (84.1%)	2,300 (85.1%)	2,200 (84.0%)
Male	1,225 (15.6%)	403 (15.9%)	403 (14.9%)	419 (16.0%)
Age (years)	31	31	31	31
Education				
Grades 1 to 7 (primary school)	1,744 (22.3%)	558 (22.1%)	651 (24.2%)	535 (20.5%)
Grades 8 to 12 (secondary school)	5,612 (71.7%)	1,790 (71.0%)	1,906 (70.8%)	1,916 (73.4%)
Higher education	327 (4.2%)	112 (4.4%)	89 (3.3%)	126 (4.8%)
No education	140 (1.8%)	62 (2.5%)	45 (1.7%)	33 (1.3%)
Missing	33	12	12	9
Wealth^b^				
Wealthiest quintile	910 (11.8%)	287 (11.5%)	234 (8.9%)	389 (15.1%)
Second wealthiest quintile	1,419 (18.4%)	416 (16.7%)	391 (14.8%)	612 (23.7%)
Intermediate quintile	1,384 (17.9%)	360 (14.4%)	424 (16.0%)	600 (23.3%)
Second poorest quintile	1,521 (19.7%)	453 (18.2%)	569 (21.5%)	499 (19.3%)
Poorest quintile	2,483 (32.2%)	979 (39.2%)	1,024 (38.8%)	480 (18.6%)
Missing	139	39	61	39
HIV care cascade				
Unaware of status	3,866 (51.2%)	1,350 (55.9%)	1,197 (46.4%)	1,319 (51.7%)
Aware of status, but not in care	819 (10.9%)	261 (10.8%)	294 (11.4%)	264 (10.3%)
In care, but not on ART	345 (4.6%)	107 (4.4%)	111 (4.3%)	127 (5.0%)
Used ART less than 5 years	1,872 (24.8%)	526 (21.8%)	751 (29.1%)	595 (23.3%)
Used ART 5 or more years	643 (8.5%)	170 (7.0%)	226 (8.8%)	247 (9.7%)
Missing	311	120	124	67
Language(s) used for the questionnaire				
English and Afrikaans	149 (1.9%)	38 (1.5%)	87 (3.2%)	24 (0.9%)
English and Bemba	1,948 (24.8%)	482 (19.0%)	796 (29.4%)	670 (25.6%)
English and Lozi	160 (2.0%)	143 (5.6%)	8 (0.3%)	9 (0.3%)
English and Nyanja	1,067 (13.6%)	413 (16.3%)	408 (15.1%)	246 (9.4%)
English and Tonga	252 (3.2%)	66 (2.6%)	25 (0.9%)	161 (6.1%)
English and Xhosa	2,373 (30.2%)	924 (36.5%)	721 (26.7%)	728 (27.8%)
English only	1,907 (24.3%)	468 (18.5%)	658 (24.3%)	781 (29.8%)
Health-related quality of life score	0.884	0.886	0.884	0.881
Mobility				
No problems walking around	7,611 (96.9%)	2,469 (97.4%)	2,625 (97.1%)	2,517 (96.1%)
Any problems walking around	245 (3.1%)	65 (2.6%)	78 (2.9%)	102 (3.9%)
Self-care				
No problems washing and dressing	7,635 (97.2%)	2,482 (97.9%)	2,621 (97.0%)	2,532 (96.7%)
Any problems washing and dressing	221 (2.8%)	52 (2.1%)	82 (3.0%)	87 (3.3%)
Daily activities				
No problems doing daily activities	7,521 (95.7%)	2,437 (96.2%)	2,588 (95.7%)	2,496 (95.3%)
Any problems doing daily activities	335 (4.3%)	97 (3.8%)	115 (4.3%)	123 (4.7%)
Pain/discomfort				
No problems with pain/discomfort	7,007 (89.2%)	2,304 (90.9%)	2,407 (89.0%)	2,296 (87.7%)
Any problems with pain/discomfort	849 (10.8%)	230 (9.1%)	296 (11.0%)	323 (12.3%)
Anxiety/depression				
No problems with anxiety/depression	7,206 (91.7%)	2,299 (90.7%)	2,477 (91.6%)	2,430 (92.8%)
Any problems with anxiety/depression	650 (8.3%)	235 (9.3%)	226 (8.4%)	189 (7.2%)

a Statistics presented: N (%); mean age and health-related quality of life score.

b Wealth was measured relative to the full HPTN 071 (PopART cohort).

The prevalence of most socio-demographic and clinical characteristics was similar between the trial arms. However, there were differences between the arms in wealth, language used for the questionnaire and the HIV care cascade (Table 1).

At baseline, HRQoL scores also differed slightly between the trial arms (Arm A: 0.886, Arm B: 0.884, Arm C: 0.881). Among the HRQoL dimensions, problems were most often reported with pain/discomfort (10.8%) and anxiety/depression (8.3%). The prevalence of problems was higher in Arm C than Arms A and B for most HRQoL dimensions, although more individuals reported problems with anxiety/depression in Arm A than Arms B and C (Table 1).

### 3.3 HRQoL score

After 36 months, the mean HRQoL score was 0.892 (95% CI: 0.887–0.898) in Arm A, 0.886 (0.877–0.894) in Arm B and 0.888 (0.884–0.892) in Arm C. In adjusted analyses, there was no evidence of a difference in HRQoL score between Arms A and C (aMD: 0.003, -0.001-0.006), between Arms B and C (-0.004, -0.014-0.005) or between Arms A + B and C (0.001, -0.003-0.004). There was also no difference in HRQoL scores for the sex subgroup analyses (Fig. 2, Table A.1).

### 3.4 HRQoL dimensions

At endpoint, PLHIV reported more problems with the dimensions of pain/discomfort (prevalence: 7.5%) and anxiety/depression (4.8%) than with the dimensions of mobility (2.1%), daily activities (2.8%) or self-care (1.3%).

There was evidence of a lower prevalence of problems with pain/discomfort in Arm A than C (aPR: 0.37, 0.14–0.97), but there was no evidence for a difference for any other arm comparison (Fig. 3, Table A.2-A.6). The effect was also present in the sex subgroup analyses (women: 0.38, 0.15–0.97; men: 0.41, 0.20–0.83).

### 3.5 12-month and 24-month outcomes

**Fig. 4:**
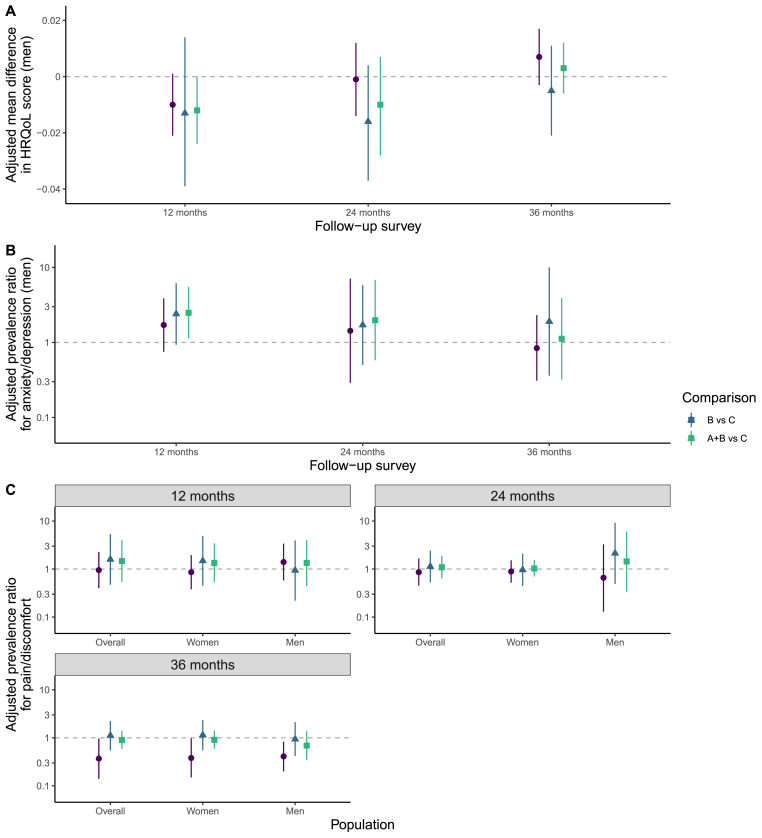
Effect of universal testing and treatment over time (analyses with evidence of a difference) A) Adjusted estimates of the mean difference in HRQoL score between the arms for men. B) Adjusted estimates of prevalence ratios for problems with anxiety/depression for men. C) Adjusted estimates of prevalence ratios for problems with pain/discomfort overall, for women and for men. Overall estimates were adjusted for age, sex, language(s) used, baseline wealth, baseline position in the HIV care cascade and baseline HRQoL score or prevalence of problems in each HRQoL dimension. Analyses stratified by sex were adjusted for the same variables, except sex was excluded. Prevalence ratios less than one indicate fewer problems under the intervention. A log scale is used for prevalence ratios. Error bars: 95% confidence intervals.

At 12 months, there was evidence of lower HRQoL (aMD: -0.012, -0.024-0) and increased problems with anxiety/depression (aPR: 2.48, 1.13–5.47) among men when Arms A + B was compared with Arm C. At 24 months, there was no evidence of a difference between arms in any HRQoL outcome (Fig. 4, Fig. A.4-A.7).

### 3.6 Exploratory analyses

Including people who seroconverted did not substantially alter the estimated coefficients, although the effects reported above were no longer statistically significant (appendix section 2, table A.7-A.12). In analyses stratified by ART status, there was evidence of higher HRQoL (aMD: 0.004, 0–0.008) and fewer problems with pain/discomfort (aPR: 0.30, 0.09–0.96) among women who were not on ART when Arm A was compared with Arm C (appendix section 2, Table A.13-A.24).

## 4 Discussion

In this study we did not find that the HPTN 071 (PopART) intervention was associated with a change in overall HRQoL at 36 months among adults living with HIV at baseline. However, communities that received the full intervention had a lower prevalence of problems with pain/discomfort at the endpoint. Additionally, at 12 months there was evidence of lower overall HRQoL scores and increased anxiety/depression among men when comparing the pooled data from Arms A and B with control communities.

This study has several strengths. Firstly, it uses data from 21 urban and peri-urban communities in two countries with different socio-economic contexts, which means that findings may be generalisable to diverse urban settings in Southern Africa (Hayes et al., 2019). In addition, the HRQoL information is from a community sample, rather than a clinic-based group, reducing bias towards PLHIV who seek healthcare (Thomas et al., 2017). Thirdly, data were collected from randomly sampled adults to improve representativeness, and the study had a large sample size which may increase precision. Data were also gathered on a wide variety of topics, allowing adjustment for a range of relevant confounders. Finally, robust methods were applied to detect associations while accounting for the study design (Hayes & Moulton, 2017).

Our finding that the intervention was not associated with improved overall HRQoL in the general population of PLHIV contrasts with results from a large multi-country study among PLHIV initiating treatment (Lifson et al., 2017). The multi-country study included analysis of two measures of overall HRQoL: perceived current health assessed with a standalone visual analogue scale and general health perception assessed with the Short-Form 12-Item Health Survey version 2 (De Boer et al., 2004; Ware et al., 1996). The study reported that universal treatment improved both measures, which differs from the results reported here (Lifson et al., 2017). Nonetheless, our finding is in line with studies indicating limited effects of interventions that included UTT on some outcomes linked to wellbeing, including HIV stigma and living standards (Stangl et al., 2020; Steinert et al., 2020). Moreover, our finding of an effect of the intervention on pain/discomfort parallels results from three randomised trials of universal treatment, which all reported improvements in HRQoL dimensions measuring pain (Bunupuradah et al., 2013; Kumar et al., 2021; Lifson et al., 2017).

Many PLHIV had high HRQoL scores at baseline and this may help to explain why the intervention was not associated with an increase in HRQoL score in most analyses. When a cohort is relatively well, and HRQoL scores are already high, potential for improvement is small (Davis & Pathak, 2001). It is unclear why this cohort had high baseline HRQoL scores, but other studies have reported similar findings (Lifson et al., 2017; Osei-Yeboah et al., 2017; Yaya et al., 2019). One possible explanation is access to treatment. From the start of the trial, most PLHIV with more advanced HIV infections were accessing treatment, which largely prevented severe symptoms. The majority of PLHIV who were not on treatment were individuals who were at the early stages of infection and so were experiencing no symptoms, or mild symptoms. As a result, the population of PLHIV may have been comparatively healthy, with high HRQoL and limited opportunity for further HRQoL gains. Consequently, it might be suggested that resources for improving HRQoL should be focused on other settings with greater ability to benefit. However, the lack of an association between the intervention and overall HRQoL also indicates that, in this setting, further improving HRQoL among PLHIV will require more than enhanced testing and treatment. Other approaches, especially those that involve PLHIV in identifying challenges to wellbeing and responding, could be more effective (Andersson et al., 2020). The limited effect of the intervention on HRQoL also has consequences for economic analyses; it indicates that economic evaluations of UTT that use mortality impact measures are sufficient, and complex measures that combine morbidity and mortality impacts, such as Quality Adjusted Life Years, may not be needed.

Although this study did not detect a large increase in overall HRQoL associated with the intervention, neither was there strong evidence for negative effects at 36 months. This implies that any negative effects of UTT are small or transient, which aligns with some existing longitudinal evidence (Kiene et al., 2018; Tomita et al., 2014). For instance, in a study of newly diagnosed Ugandan PLHIV, depressive symptoms initially rose following diagnosis, but then rapidly decreased, falling below the cut-off for possible depression after 15 days, on average (Kiene et al., 2018). Notably, we found no evidence of a difference in anxiety/depression between trial arms at 24 or 36 months. Moreover, the lower HRQoL scores and increased anxiety/depression in Arms A + B compared to Arm C among men at 12 months may reflect transient negative consequences of UTT. The change to universal ART in Arm B happened during the 12-month survey in Zambia, while in South Africa, the CD4 count threshold for initiating treatment rose to 500 cells/mm^3^ immediately before the survey, which may have affected HRQoL. Policy changes also occurred in Arm C, but without universal testing the effect may have been smaller. In terms of implications for policy, the findings of this study, along with those from previous studies, indicate that expansion of UTT should not be constrained because of concerns around lasting HRQoL losses, while also highlighting the need to fully investigate trends in HRQoL immediately after UTT introduction.

Further support for expansion of UTT is provided by the finding that there was a lower prevalence of problems with pain/discomfort in Arm A communities than in Arm C communities after 36 months, with similar effects among men and women. As no effect was found in analyses focused on Arm B or Arms A + B, differences were likely driven by earlier access to universal ART (Hayes et al., 2019). It is important to maximise any improvement in pain among PLHIV as it is commonly under-treated (Parker et al., 2014). However, there has been limited research on lessening pain in the ART era, so confirming these findings in other settings may be useful (Sabin et al., 2018).

This study has limitations. Some intervention and control communities were geographically close, which may have reduced the intervention effect as PLHIV could receive services in neighbouring communities (Hayes et al., 2014). Additionally, men were under-represented in the cohort; this could have led to selection bias in our estimates. Also, a Zambian version of the EQ-5D-5L did not exist, so the study team translated it, which may have resulted in small alterations in interpretation. Moreover, the primary outcome of HPTN 071 (PopART) was not HRQoL, and power to detect small changes in HRQoL was limited. Equally the analysis involved many significance tests, which can increase the probability of a false positive finding due to chance. Lastly, although many possible confounders were considered, some may have been unobserved and could have affected results if they differed systematically by arm.

## 5 Conclusions

In the primary analysis of HPTN 071 (PopART), UTT was found to have reduced the population-level incidence of HIV infection (Hayes et al., 2019). However, in this secondary analysis of data from HPTN 071 (PopART), we did not find evidence of an association between UTT and HRQoL. Our results suggest that resources for raising HRQoL should be focused on settings where larger increases in HRQoL may be possible, but also indicate that, within the study context, strategies other than UTT may be required to raise HRQoL. Nonetheless, negative effects of the intervention on overall HRQoL at 36 months were not detected, implying that UTT roll-out should not be constrained because of concerns around lasting HRQoL losses. In addition, the full intervention was associated with reduced pain/discomfort, which is an important potential benefit of UTT. There was also some evidence of a transient decrease in HRQoL among men following introduction of improved access to ART, underlining the need to understand how UTT affects HRQoL across shorter timescales.

## Ethical statement

Ethical approval for the HPTN 071 (PopART) trial was provided by institutional review boards at the London School of Hygiene and Tropical Medicine (ref: 6326), Stellenbosch University (ref: N12/11/074), and the University of Zambia (ref’s: HPTN071/PopART UNZA BREC REF:-011-11-12, HPTN071a/Stigma-001-02-14, HPTN071-2/Phylogenetics UNZA BREC REF:-004-05-15). Participants provided written informed consent.

## Data sharing

The HPTN 071 (PopART) data sharing policy is included in the appendix (section 5).

## Disclaimer

The content is solely the responsibility of the authors and does not necessarily represent the official views of the NIAID, NIMH, NIDA, PEPFAR, 3ie, the Bill & Melinda Gates Foundation, NIHR, Public Health England or the Department of Health and Social Care.

For the purpose of open access, the author has applied a CC BY public copyright licence to any Author Accepted Manuscript version arising from this submission.

## CRediT authorship contribution statement

**Katherine Davis:** Conceptualization, Validation, Formal analysis, Writing – original draft, Writing – review & editing, Visualization. **Michael Pickles:** Conceptualization, Formal analysis, Writing – review & editing, Supervision. **Simon Gregson:** Conceptualization, Formal analysis, Writing – review & editing, Supervision. **James R. Hargreaves:** Investigation, Writing – review & editing. **Helen Ayles:** Investigation, Writing – review & editing, Supervision. **Peter Bock:** Investigation, Writing – review & editing, Supervision. **Triantafyllos Pliakas:** Investigation, Writing – review & editing. **Ranjeeta Thomas:** Methodology, Writing – review & editing. **Julius Ohrnberger:** Methodology, Writing – review & editing. **Justin Bwalya:** Investigation, Writing – review & editing. **Nomtha Bell-Mandla:** Investigation, Writing – review & editing. **Kwame Shanaube:** Investigation, Writing – review & editing. **William Probert:** Investigation, Writing – review & editing. **Graeme Hoddinott:** Investigation, Writing – review & editing. **Virginia Bond:** Investigation, Writing – review & editing. **Richard Hayes:** Investigation, Formal analysis, Writing – review & editing, Supervision. **Sarah Fidler:** Investigation, Writing – review & editing, Supervision. **Katharina Hauck:** Conceptualization, Validation, Methodology, Writing – review & editing, Supervision.

## Declaration of competing interest

The authors declare the following financial interests/personal relationships which may be considered as potential competing interests: KH received professional fees for advisory activities for Pfizer and GlaxoSmithKline for research unrelated to this study, and professional fees from the World Health Organization for research unrelated to this study. SG declares holding shares in AstraZeneca and GlaxoSmithKline.All other authors declare no competing interests.

## Data Availability

The HPTN 071 (PopART) data sharing policy is included in the appendix (section 5).
