# Supplementary material for: The effect of universal testing and treatment for HIV on health-related quality of life – An analysis of data from the HPTN 071 (PopART) cluster randomised trial
Source: SSM Popul Health. 2023 Jul 22;23:101473. doi: 10.1016/j.ssmph.2023.101473 (PMC10413193; doi:10.1016/j.ssmph.2023.101473)
Supplement: Multimedia component 1 [file mmc1.pdf]

Appendix

1. HPTN 071 (PopART) - full description 4
Trial design and randomisation 4
Interventions 4
Participants 4
Outcome measurement – HRQoL 5
Assessment of wealth 5
Trial end 5
Effect of the intervention on other outcomes 5
2. Exploratory analyses 7
Inclusion of people who seroconverted during the trial 7
Subgroup analyses stratified by ART status at endpoint 7
3. Supplementary figures and tables 8
Figure A.1. Location of HPTN 071 (PopART) communities (clusters) in Zambia and South Africa, with colour coded triplets. 9
Figure A.2. Timeline for HPTN 071 (PopART) showing data collection periods and Antiretroviral Therapy (ART) eligibility. 10
Figure A.3. Summary of HPTN 071 (PopART) study design and the cohort that was analysed. 11
Figure A.4. Effect of universal testing and treatment on HRQoL score in people living with HIV at 12 months 12
Figure A.5. Effect of universal testing and treatment on dimensions of HRQoL in people living with HIV at 12 months 13
Figure A.6. Effect of universal testing and treatment on HRQoL score in people living with HIV at 24 months 14
Figure A.7. Effect of universal testing and treatment on dimensions of HRQoL in people living with HIV at 24 months 15
Table A.1. Effect of the universal testing and treatment intervention on health-related quality of life score among people living with HIV at 36 months. 16
Table A.2. Effect of the universal testing and treatment intervention on problems with mobility at 36 months 17
Table A.3. Effect of the universal testing and treatment intervention on problems with self-care at 36 months 18
Table A.4. Effect of the universal testing and treatment intervention on problems with daily activities at 36 months 19
Table A.5. Effect of the universal testing and treatment intervention on problems with pain/discomfort at 36 months 20
Table A.6. Effect of the universal testing and treatment intervention on problems with anxiety/depression at 36 months. 21

Table A.7. Adjusted effect of the universal testing and treatment intervention on health-related quality of life score at 36 months, when people who seroconverted during the trial were included. 22

Table A.8. Adjusted effect of the universal testing and treatment intervention on problems with dimensions of health-related quality of life at 36 months, when people who seroconverted during the trial were included. 23

Table A.9. Adjusted effect of the universal testing and treatment intervention on health-related quality of life score at 12 months, when people who seroconverted during the trial were included. 24

Table A.10. Adjusted effect of the universal testing and treatment intervention on problems with dimensions of health-related quality of life at 12 months, when people who seroconverted during the trial were included. 25

Table A.11. Adjusted effect of the universal testing and treatment intervention on health-related quality of life score at 24 months, when people who seroconverted during the trial were included. 26

Table A.12. Adjusted effect of the universal testing and treatment intervention on problems with dimensions of health-related quality of life at 24 months, when people who seroconverted during the trial were included. 27

Table A.13. Adjusted effect of the universal testing and treatment intervention on health-related quality of life score at 36 months, among people who were unaware of their status, or aware of their status but not on antiretroviral therapy. 28

Table A.14. Adjusted effect of the universal testing and treatment intervention on problems with dimensions of health-related quality of life at 36 months, among people who were unaware of their status, or aware of their status but not on antiretroviral therapy. 29

Table A.15. Adjusted effect of the universal testing and treatment intervention on health-related quality of life score at 12 months, among people who were unaware of their status, or aware of their status but not on antiretroviral therapy. 30

Table A.16. Adjusted effect of the universal testing and treatment intervention on problems with dimensions of health-related quality of life at 12 months, among people who were unaware of their status, or aware of their status but not on antiretroviral therapy. 31

Table A.17. Adjusted effect of the universal testing and treatment intervention on health-related quality of life score at 24 months, among people who were unaware of their status, or aware of their status but not on antiretroviral therapy. 32

Table A.18. Adjusted effect of the universal testing and treatment intervention on problems with dimensions of health-related quality of life at 24 months, among people who were unaware of their status, or aware of their status but not on antiretroviral therapy. 33

Table A.19. Adjusted effect of the universal testing and treatment intervention on health-related quality of life score at 36 months, among people who were on antiretroviral therapy. 34

Table A.20. Adjusted effect of the universal testing and treatment intervention on problems with dimensions of health-related quality of life at 36 months, among people who were on antiretroviral therapy. 35

Table A.21. Adjusted effect of the universal testing and treatment intervention on health-related quality of life score at 12 months, among people who were on antiretroviral therapy. 36

Table A.22. Adjusted effect of the universal testing and treatment intervention on problems with dimensions of health-related quality of life at 12 months, among people who were on antiretroviral therapy. 37

Table A.23. Adjusted effect of the universal testing and treatment intervention on health-related quality of life score at 24 months, among people who were on antiretroviral therapy. 38

Table A.24. Adjusted effect of the universal testing and treatment intervention on problems with dimensions of health-related quality of life at 24 months, among people who were on antiretroviral therapy. 39

4. References 40

5. HPTN 071 (PopART) data sharing policy 42

1. HPTN 071 (PopART) - full description

Trial design and randomisation

HPTN 071 (PopART) was a three-arm matched cluster randomised controlled trial (C-RCT), conducted between 2013 and 2018, which assigned 21 urban and peri-urban communities in Zambia and South Africa to seven triplets, matched by geographic location and estimated HIV prevalence. Each community was the catchment population of a government clinic (Hayes et al., 2014, 2019). In total, the seven triplets had a population of approximately one million (Hayes et al., 2019). Communities in each triplet were randomly assigned to three trial groups in simultaneous public ceremonies. Restricted randomisation was used to assign communities within triplets to one of three study arms, while maintaining balance across the trial groups in population size, baseline antiretroviral therapy (ART) coverage and HIV prevalence (Vermund et al., 2013).

Interventions

Arm A communities received the full combination prevention intervention, with rapid HIV testing and universal ART for people living with HIV (PLHIV). Community health workers visited homes to provide a range of services, including:

- HIV education and prevention information
- HIV rapid testing
- Referral of PLHIV to care for initiation on ART
- Support with linkage and retention in HIV care for PLHIV
- Referral of HIV-negative men to voluntary medical male circumcision services
- Referral of pregnant women living with HIV to antenatal care
- Referral to reproductive health and family planning services
- Tuberculosis symptom screening followed by collection of sputum samples and referral to the health facility for those diagnosed
- Sexually transmitted infection screening and referral to care for those with symptoms
- Condom provision
- Community engagement, such as male health fairs, development of youth friendly corners at clinics and short-term “zonal” campaigns in some areas

Arm B communities received the same intervention, but ART was provided according to local guidelines. Initially this meant that ART was only provided to individuals whose CD4 count was below a national threshold value; however, in 2016, local guidelines switched so that ART was provided universally. Arm C communities acted as the control and received standard care. HIV testing and treatment were available at government health facilities, which were given support to ensure that HIV services met the requirements of local guidelines, as far as was possible (Hayes et al., 2019).

Participants

To measure the effect of the combination prevention intervention, a Population Cohort (PC) was used (Hayes et al., 2019). Members of the PC were selected via random sampling in Arm A, Arm B and Arm C communities. Specifically, within randomly sampled households, one adult resident aged 18–44 years was selected at random for the PC. Members of the PC completed assessment surveys with field research assistants at baseline between 2013 and 2015, as well as at 12, 24 and 36 months (Hayes et al., 2014). Additional individuals were recruited at 12 months and 24 months, from households that had not previously been selected, in communities where recruitment targets had not been met. The baseline participation rate among eligible individuals was 82.5%; discontinuation rates were 13.5% at 12 months, 13.2% at 24 months and 27.8% at 36 months (Hayes et al., 2019).

For this study, a cohort of PLHIV who were present at baseline were selected from within the larger PC. PLHIV were identified based on HIV testing results. The HIV testing procedure involved a single fourth-generation test, with laboratory confirmation for quality control and to identify incident infections. The cohort of PLHIV was restricted to participants who had chosen to respond to all the HRQoL questions during at least one survey. Where there was item non-response for the HRQoL questions during one or more surveys, but not all of them, PLHIV were temporarily excluded from the cohort for the survey rounds where they had not responded to the HRQoL questions. For example, if an individual did not answer the HRQoL questions during the 12-month survey, but did respond at all other surveys,

they were not included in the cohort for analyses focused on outcomes at 12 months, but their data was still used in analyses of outcomes at 24 and 36 months. This maximised the number of participants with data available for analysis.

In addition to choosing not to answer the HRQoL questions during a survey, participants within the larger PC could leave the study altogether or skip a survey and return later. This was reflected in the cohort of PLHIV, with attrition occurring over time as well as individuals leaving, and then returning to, the cohort.

Outcome measurement – HRQoL

The EuroQol five dimensions, five levels questionnaire (EQ-5D-5L) was used to measure HRQoL. In South Africa it was possible to use certified translations of the EQ-5D-5L, which were developed through cultural adaptation procedures including multiple forwards and backwards translations, pilot testing with native speakers and interaction with local teams working on measuring health-related quality of life (Rabin et al., 2014). Certified translations were not available for Zambia, so the study team, which included native speakers, carefully translated the questions into local dialects (Thomas et al., 2017).

The EQ-5D-5L was chosen to measure HRQoL for several reasons. Firstly, although the EQ-5D-5L was originally developed in European populations, it is now in regular use globally, including in sub-Saharan African populations (Herdman et al., 2011; Kastien-Hilka et al., 2017; Lartey et al., 2019; Rabin et al., 2014; Thomas et al., 2017). Secondly, there is previous experience of using the EQ-5D-5L in PLHIV in a variety of settings and it has well established reliability and validity (Miners et al., 2014; Thomas et al., 2017; Tran et al., 2012; Wu et al., 2013).

To assess overall HRQoL, responses across the five questions in the EQ-5D-5L were characterised with a single score. A pre-existing mapping of EQ-5D-5L responses to scores, from EuroQoL, was used to do this. With the mapping, it is possible to read across from any set of five EQ-5D-5L responses to a score. Mappings of this type are known as a valuation of health states, and they usually fit EQ-5D-5L responses to scores measured on an interval scale from 1 (best) to 0 (worst, equivalent to death). Valuations vary by country and culture, so a local valuation from a general population sample was required for this study.(Andrade et al., 2020; Pickard et al., 2019; Welie et al., 2020) Time-trade-off, visual analogue scale and discrete choice experiments can be used to elicit valuations; however, very few experiments to determine valuations have been performed in sub-Saharan African countries (Devlin & Brooks, 2017; Oppe et al., 2014; Yang et al., 2022). At the time of this study no valuations were available for Zambia, so valuations from Zimbabwe were used (Van Hout et al., 2012). The valuations reflect local beliefs about health and consequently, the best health state, where individuals report no problems, is given a score of 0.9 rather than 1, and the lowest scores are below zero (a state worse than death) in the Zimbabwean valuations (Janssen et al., 2013; Van Hout et al., 2012). For this analysis, values less than or equal to zero were recoded to 0.001.

Assessment of wealth

During the survey participants were asked about ownership of assets. A principal components analysis of the data from these questions was used to generate an index capturing the relative wealth of participants (Hargreaves et al., 2018).

Trial end

The trial stopped after the 36 months of follow-up required had been completed.

Effect of the intervention on other outcomes

Previous analysis has examined how HIV incidence, viral load suppression, knowledge of HIV status among PLHIV and ART uptake among PLHIV varied across the trial arms (Hayes et al., 2019).

HIV incidence in the PC between months 12 and 36 was 1.5 per 100 person-years in Arm A, 1.1 per 100 person-years in Arm B, and 1.6 per 100 person-years in Arm C. When HIV incidence was compared between trial arms, there was no evidence of a difference between Arm A and Arm C (adjusted rate ratio: 0.93, 95% confidence interval: 0.74-1.18), although there was evidence of lower HIV incidence in Arm B compared with Arm C (0.70, 0.55-0.88) and Arms A+B compared with Arm C (0.81, 0.66-0.99).

The percentage of PLHIV in the PC who were virally suppressed at 24 months was 71.9% in Arm A, 67.5% in Arm B, and 60.2% in Arm C. Comparisons of the prevalence of viral suppression between trial arms revealed that there

was evidence for greater viral suppression in Arm A than Arm C (adjusted prevalence ratio: 1.16, 95% confidence interval: 0.99-1.36) , but not in Arm B than Arm C (1.08, 0.92-1.27).

The estimated percentage of PLHIV who know their HIV status at 36 months, based on data collected by community health workers in intervention arms, was 91.3% in Arm A, and 90.8% in Arm B.

The estimated percentage of PLHIV who were on ART at 36 months, based on data collected by community health workers in intervention arms, was 81% in Arm A, and 80% in Arm B.

2. Exploratory analyses

Inclusion of people who seroconverted during the trial

The methods for the analyses that included people who seroconverted were the same as the methods for the main analyses, except that the variable capturing uptake of HIV care at baseline was expanded to have a category for people who were HIV-negative.

The adjusted mean differences (aMDs) and adjusted prevalence ratios (aPRs) from the analyses that included people who seroconverted were generally similar to those from the main analysis (Table A.7-A.12). However, the only statistically significant result was a higher prevalence of anxiety/depression among women at 36 months, when Arm B was compared with Arm C (3.48, 1.00-12.08). The effect of the intervention on pain/discomfort at 36 months when Arm A was compared with Arm C was no longer statistically significant for the overall population (aPR: 0.38, 95% confidence interval, CI: 0.14-1.03), among women (0.40, 0.15-1.06), or among men (0.39, 0.14-1.10). Similarly, at 12 months, there was not evidence of a difference in overall HRQoL (aMD: -0.011, -0.024-0.001) or anxiety/depression (aPR: 2.31, 0.91-5.87) among men, when Arms A+B was compared with Arm C.

Subgroup analyses stratified by ART status at endpoint

Two alterations were made to the methods used in the main analysis for the subgroup analyses stratified by ART status. Firstly, separate analyses for men were not performed for HRQoL dimensions, because when the population was stratified, there were very few reports of problems among men in each group. Secondly, baseline position in the HIV care cascade was not adjusted for.

At 36 months, there was evidence of higher HRQoL (aMD: 0.004, 0-0.008) and lower pain/discomfort (0.30, 0.09-0.96) in Arm A compared with Arm C among women who were not on ART. There was no evidence of other differences between arms (Table A.13-A.24).

3. Supplementary figures and tables

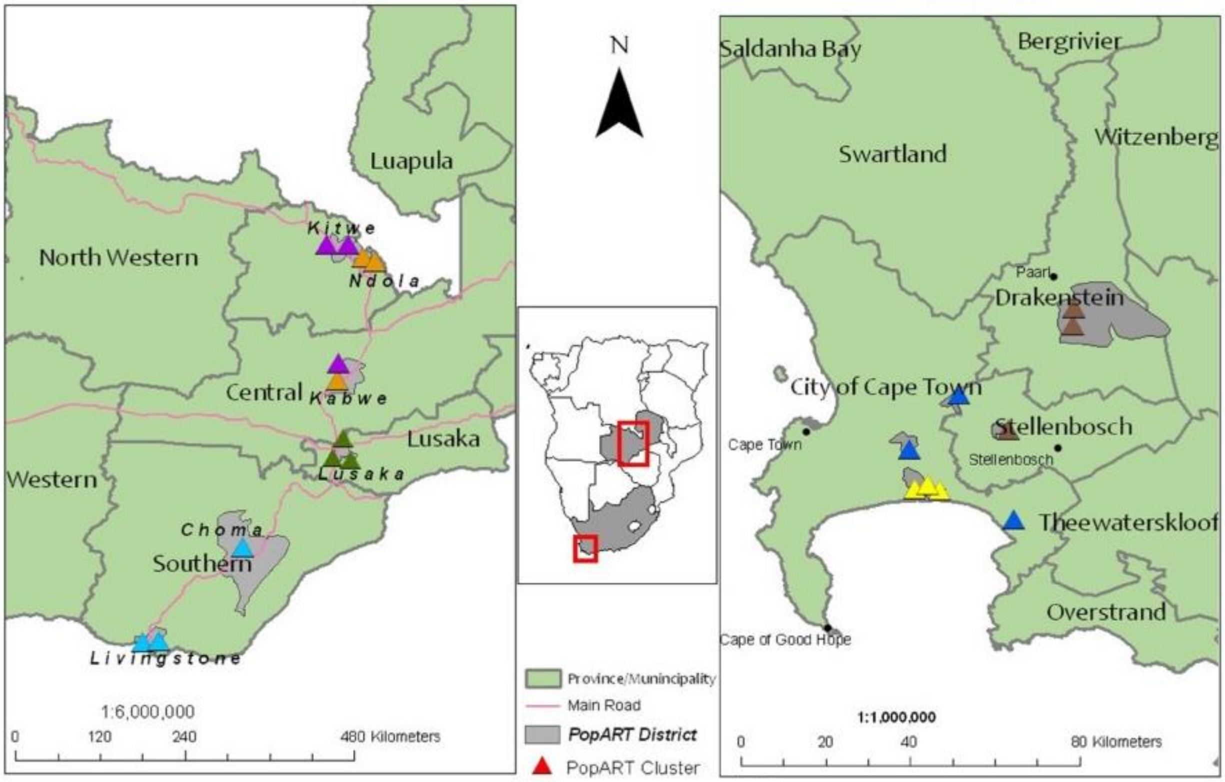

Figure A.1. Location of HPTN 071 (PopART) communities (clusters) in Zambia and South Africa, with colour coded triplets.

Zambian communities are shown in the large map on the left, and South African communities are shown in the large map on the right. The smaller central map shows the location of the study sites in the context of Southern Africa. From (Hayes et al., 2014).

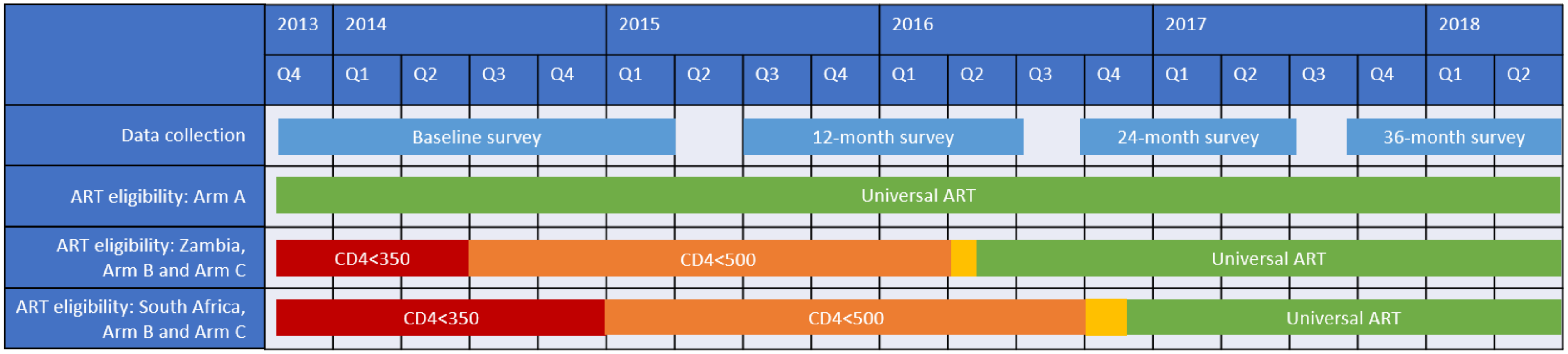

Figure A.2. Timeline for HPTN 071 (PopART) showing data collection periods and Antiretroviral Therapy (ART) eligibility.
Dates for the initiation of universal ART refer to when this was implemented in study clinics. The transition phase is shown in yellow. Adapted from (Hayes et al., 2019). Q, Quarter.

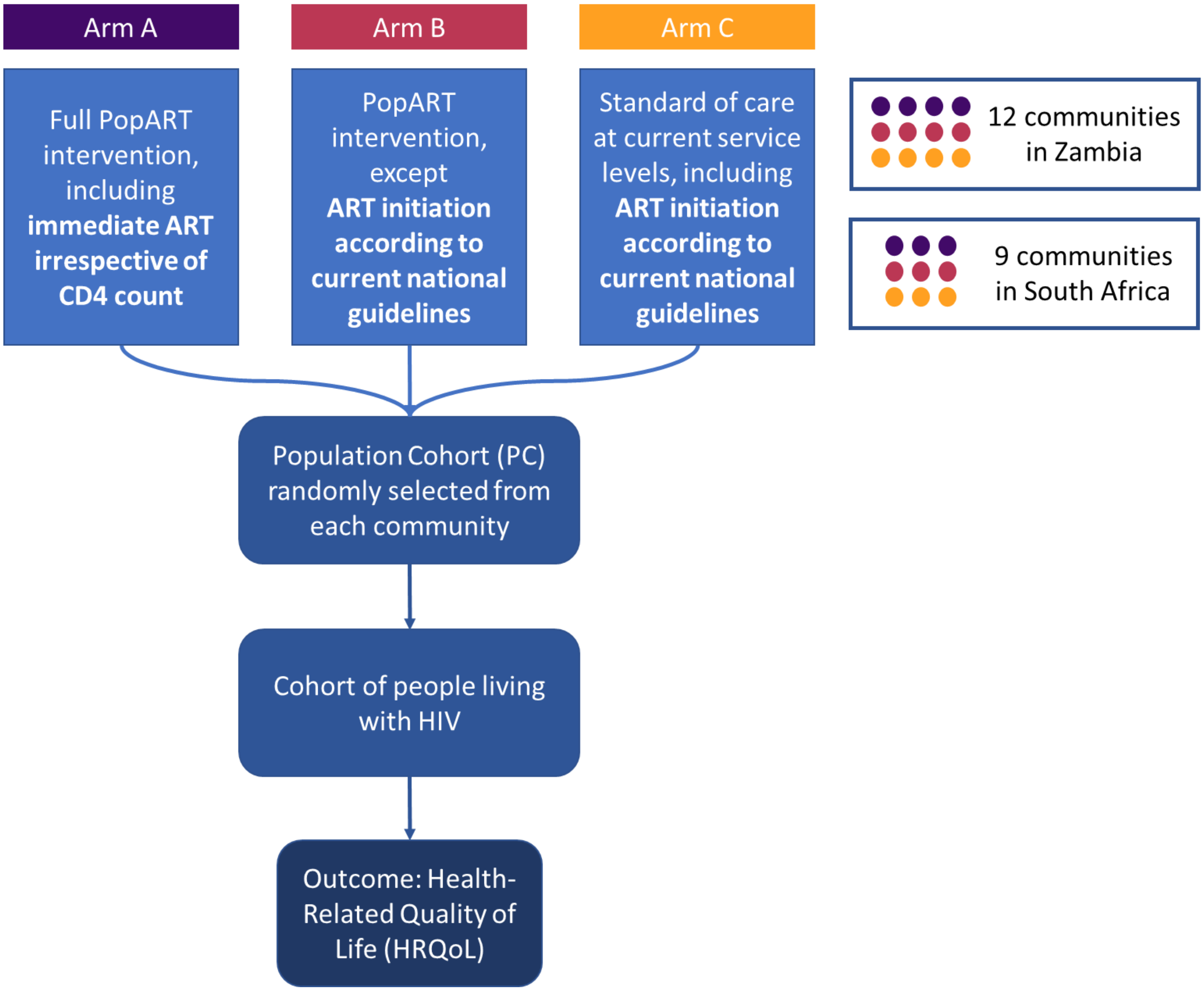

Figure A.3. Summary of HPTN 071 (PopART) study design and the cohort that was analysed. The cohort of PLHIV was selected from within the PC and were restricted to participants who had chosen to respond to all the EQ-5D-5L questions during at least one survey. Adapted from (Hayes et al., 2019).

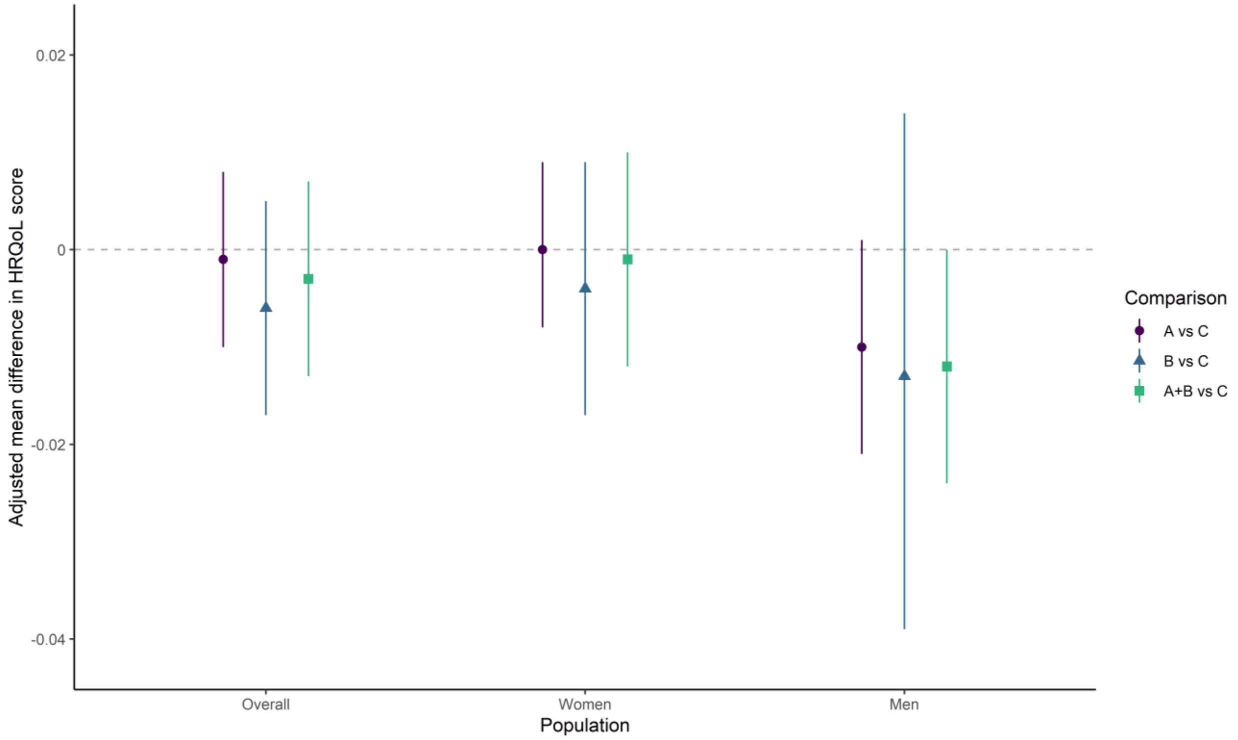

Figure A.4. Effect of universal testing and treatment on HRQoL score in people living with HIV at 12 months. Overall mean differences were adjusted for age, sex, language(s) used, baseline wealth, baseline position in the HIV care cascade and baseline HRQoL score. Analyses stratified by sex were adjusted for the same variables, except sex was excluded. Error bars: 95% confidence intervals. HRQoL: Health-related Quality of Life.

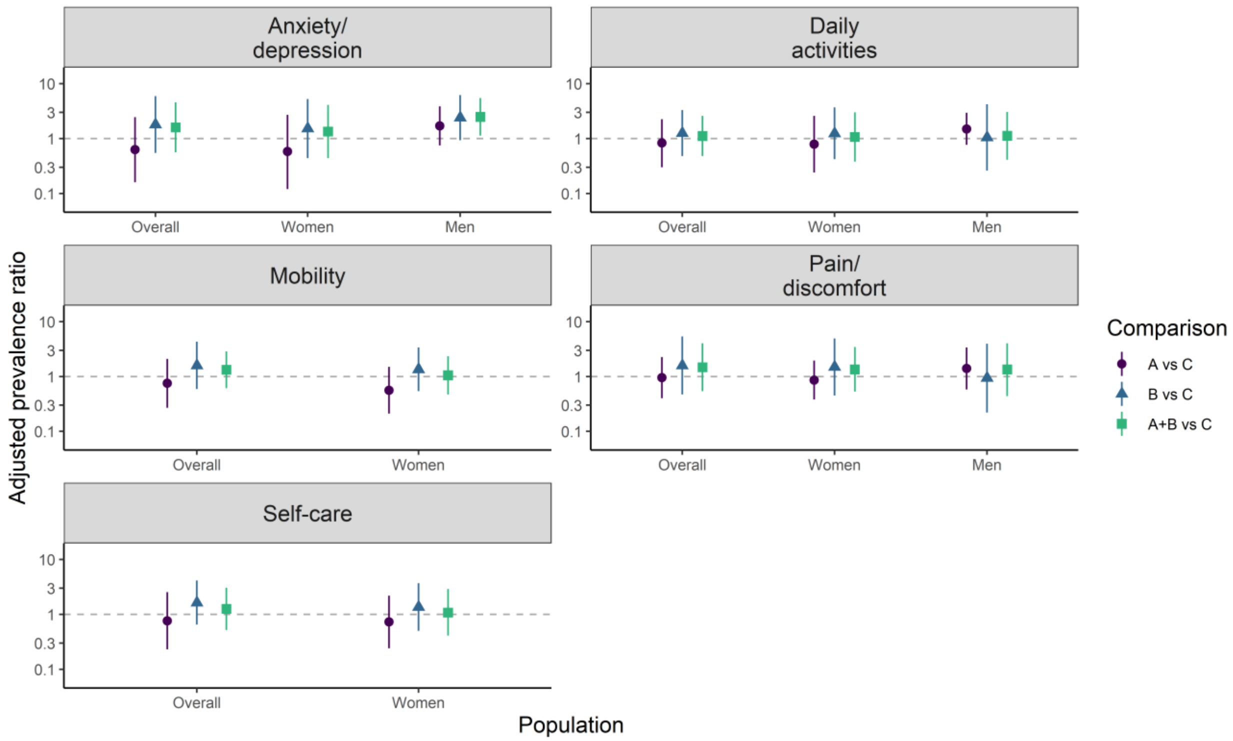

Figure A.5. Effect of universal testing and treatment on dimensions of HRQoL in people living with HIV at 12 months. Overall prevalence ratios were adjusted for age, sex, language(s) used, baseline wealth, baseline position in the HIV care cascade and baseline prevalence of problems in each HRQoL dimension. Analyses stratified by sex were adjusted for the same variables, except sex was excluded. Prevalence ratios less than one indicate fewer problems in a dimension under the intervention and a log scale is used. The very small number of men reporting problems with self-care and mobility means that estimates are not reported for these outcomes. HRQoL: Health-related Quality of Life. Error bars: 95% confidence intervals.

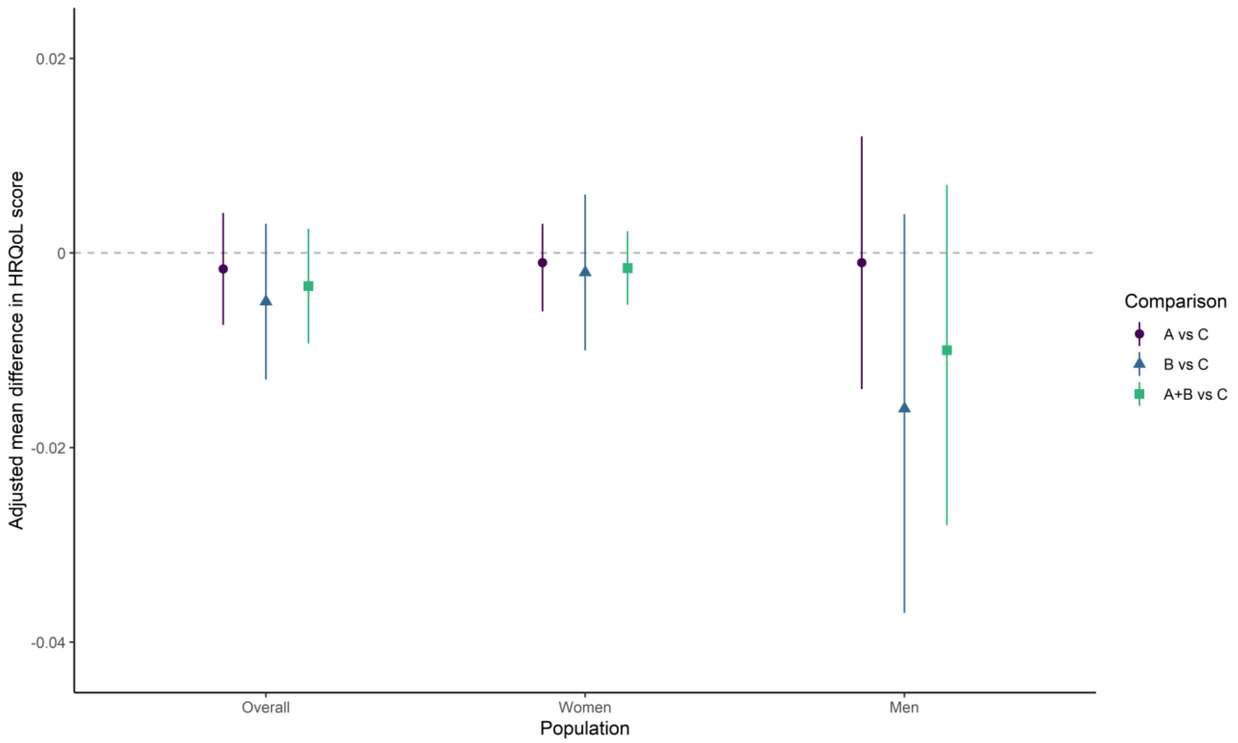

Figure A.6. Effect of universal testing and treatment on HRQoL score in people living with HIV at 24 months. Overall mean differences were adjusted for age, sex, language(s) used, baseline wealth, baseline position in the HIV care cascade and baseline HRQoL score. Analyses stratified by sex were adjusted for the same variables, except sex was excluded. Error bars: 95% confidence intervals. HRQoL: Health-related Quality of Life.

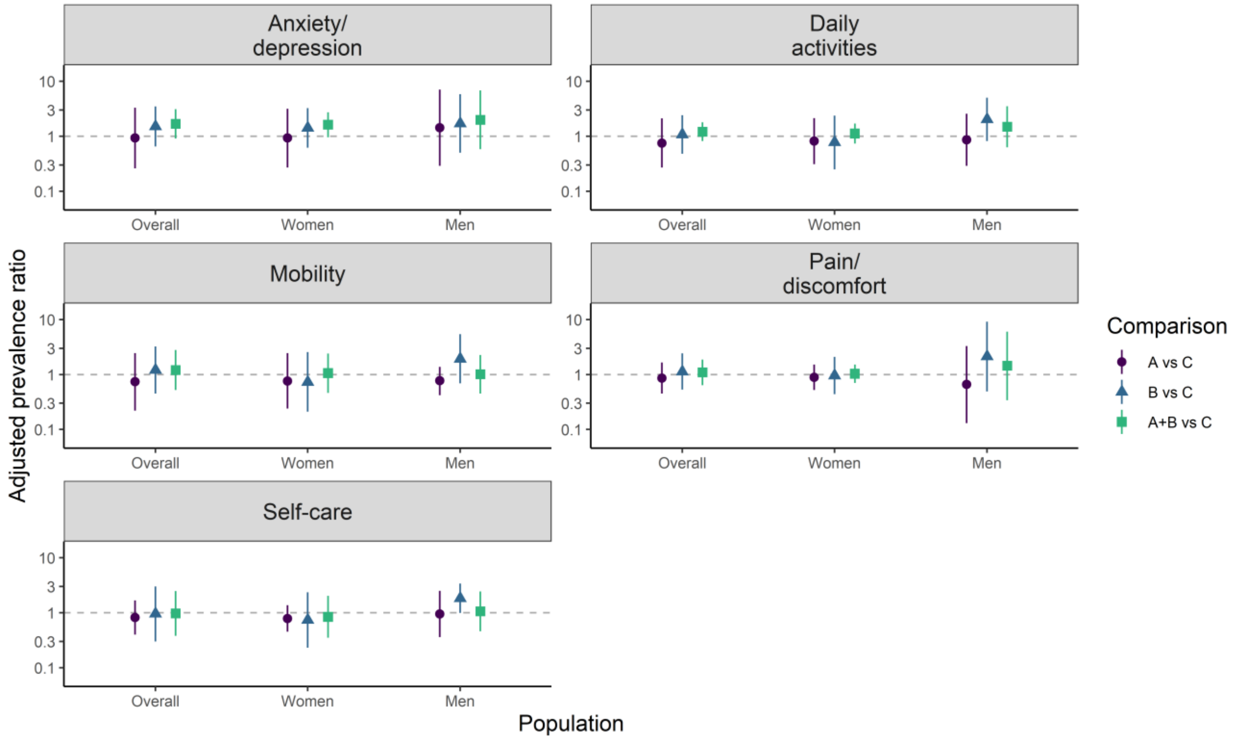

Figure A.7. Effect of universal testing and treatment on dimensions of HRQoL in people living with HIV at 24 months. Overall prevalence ratios were adjusted for age, sex, language(s) used, baseline wealth, baseline position in the HIV care cascade and baseline prevalence of problems in each HRQoL dimension. Analyses stratified by sex were adjusted for the same variables, except sex was excluded. Prevalence ratios less than one indicate fewer problems in a dimension under the intervention and a log scale is used. Error bars: 95% confidence intervals. HRQoL: Health-related Quality of Life.

Table A.1. Effect of the universal testing and treatment intervention on health-related quality of life score among people living with HIV at 36 months.

	Arm A Mean (95% CI)	Arm B Mean (95% CI)	Arm A + B Mean (95% CI)	Arm C Mean (95% CI)	Unadjusted mean differences						Adjusted mean differences					
					Arm A vs Arm C		Arm B vs Arm C		Arm A + B vs Arm C		Arm A vs Arm C		Arm B vs Arm C		Arm A + B vs Arm C	
					MD (95% CI)[1]	P-value	MD (95% CI)[1]	P-value	MD (95% CI)[1]	P-value	aMD (95% CI)[2]	P-value	aMD (95% CI)[2]	P-value	aMD (95% CI)[2]	P-value
Triplet 1	0.889 (0.883 - 0.894)	0.882 (0.874 - 0.891)	0.885 (0.880 - 0.891)	0.889 (0.886 - 0.893)	..	..	..	..	..	..	..	..	..	..	..	..
Triplet 2	0.889 (0.885 - 0.893)	0.894 (0.891 - 0.897)	0.892 (0.890 - 0.895)	0.887 (0.881 - 0.892)	..	..	..	..	..	..	..	..	..	..	..	..
Triplet 3	0.882 (0.875 - 0.889)	0.892 (0.888 - 0.895)	0.887 (0.883 - 0.891)	0.887 (0.879 - 0.894)	..	..	..	..	..	..	..	..	..	..	..	..
Triplet 4	0.893 (0.890 - 0.897)	0.894 (0.890 - 0.897)	0.894 (0.891 - 0.896)	0.893 (0.888 - 0.897)	..	..	..	..	..	..	..	..	..	..	..	..
Triplet 5	0.896 (0.894 - 0.899)	0.881 (0.876 - 0.886)	0.888 (0.885 - 0.891)	0.888 (0.883 - 0.894)	..	..	..	..	..	..	..	..	..	..	..	..
Triplet 6	0.899 (0.898 - 0.900)	0.868 (0.859 - 0.876)	0.886 (0.882 - 0.890)	0.893 (0.889 - 0.898)	..	..	..	..	..	..	..	..	..	..	..	..
Triplet 7	0.899 (0.898 - 0.900)	0.887 (0.875 - 0.900)	0.893 (0.886 - 0.900)	0.880 (0.856 - 0.900)	..	..	..	..	..	..	..	..	..	..	..	..
Overall	0.892 (0.887 - 0.898)	0.886 (0.877 - 0.894)	0.889 (0.886 - 0.893)	0.888 (0.884 - 0.892)	0.004 (-0.003 - 0.011)	0.191	-0.003 (-0.014 - 0.008)	0.572	0.001 (-0.005 - 0.007)	0.672	0.003 (-0.001 - 0.006)	0.139	-0.004 (-0.014 - 0.005)	0.311	0.001 (-0.003 - 0.004)	0.695
Women	0.892 (0.887 - 0.898)	0.886 (0.878 - 0.894)	0.890 (0.887 - 0.892)	0.888 (0.884 - 0.893)	0.004 (-0.004 - 0.012)	0.236	-0.002 (-0.013 - 0.008)	0.585	0.001 (-0.005 - 0.007)	0.639	0.002 (-0.002 - 0.006)	0.203	-0.004 (-0.013 - 0.004)	0.265	0 (-0.003 - 0.004)	0.832
Men	0.894 (0.886 - 0.900)	0.882 (0.886 - 0.897)	0.887 (0.880 - 0.895)	0.886 (0.879 - 0.893)	0.008 (-0.003 - 0.019)	0.121	-0.004 (-0.021 - 0.013)	0.582	0.002 (-0.009 - 0.012)	0.712	0.007 (-0.003 - 0.017)	0.156	-0.005 (-0.021 - 0.011)	0.486	0.003 (-0.006 - 0.012)	0.422

[1]MD (95% CI): Mean Difference (95% Confidence Interval).

[2]aMD (95% CI): adjusted Mean Difference (95% Confidence Interval). Overall mean differences were adjusted for age, sex, language(s) used, baseline wealth, baseline position in the HIV care cascade and baseline health-related quality of life score. Analyses stratified by sex were adjusted for the same variables, except sex was excluded.

Table A.2. Effect of the universal testing and treatment intervention on problems with mobility at 36 months.

	Arm A Number reporting / Total (Prevalence[1])	Arm B Number reporting / Total (Prevalence[1])	Arm A + B Number reporting / Total (Prevalence[1])	Arm C Number reporting / Total (Prevalence[1])	Unadjusted prevalence ratios						Adjusted prevalence ratios					
					Arm A vs Arm C		Arm B vs Arm C		Arm A + B vs Arm C		Arm A vs Arm C		Arm B vs Arm C		Arm A + B vs Arm C	
					PR (95% CI)[2]	P-value	PR (95% CI)[2]	P-value	PR (95% CI)[2]	P-value	aPR (95% CI)[3]	P-value	aPR (95% CI)[3]	P-value	aPR (95% CI)[3]	P-value
Overall	20 / 1,259 (1.1%)	38 / 1,381 (2.1%)	58 / 2,640 (2.0%)	24 / 1,304 (1.9%)	0.61 (0.21 - 1.77)	0.296	1.11 (0.35 - 3.48)	0.833	1.05 (0.46 - 2.42)	0.890	0.69 (0.34 - 1.40)	0.251	1.11 (0.37 - 3.36)	0.825	1.11 (0.53 - 2.29)	0.747
Women	16 / 1,088 (1.1%)	29 / 1,204 (1.7%)	45 / 2,292 (1.6%)	18 / 1,120 (1.6%)	0.65 (0.24 - 1.79)	0.336	1.03 (0.21 - 5.03)	0.962	1.01 (0.31 - 3.28)	0.982	0.76 (0.40 - 1.44)	0.338	1.06 (0.24 - 4.63)	0.928	1.09 (0.38 - 3.09)	0.850
Men	4 / 171 (2.3%)	9 / 177 (4.0%)	13 / 348 (3.1%)	6 / 184 (3.1%)	0.74 (0.21 - 2.61)	0.586	1.30 (0.44 - 3.80)	0.576	1.01 (0.33 - 3.09)	0.988	0.83 (0.23 - 2.98)	0.732	1.42 (0.61 - 3.31)	0.347	1.08 (0.44 - 2.68)	0.844

[1]Prevalence figures are calculated as the geometric means of the prevalence in each community.

[2]PR (95% CI): Prevalence Ratio (95% Confidence Interval).

[3]aPR (95% CI): adjusted Prevalence Ratio (95% Confidence Interval). Overall prevalence ratios were adjusted for age, sex, language(s) used, baseline wealth, baseline position in the HIV care cascade and baseline prevalence for each dimension. Analyses stratified by sex were adjusted for the same variables, except sex was excluded.

Table A.3. Effect of the universal testing and treatment intervention on problems with self-care at 36 months.

	Arm A Number reporting / Total (Prevalence[1])	Arm B Number reporting / Total (Prevalence[1])	Arm A + B Number reporting / Total (Prevalence[1])	Arm C Number reporting / Total (Prevalence[1])	Unadjusted prevalence ratios						Adjusted prevalence ratios					
					Arm A vs Arm C		Arm B vs Arm C		Arm A + B vs Arm C		Arm A vs Arm C		Arm B vs Arm C		Arm A + B vs Arm C	
					PR (95% CI)[2]	P-value	PR (95% CI)[2]	P-value	PR (95% CI)[2]	P-value	aPR (95% CI)[3]	P-value	aPR (95% CI)[3]	P-value	aPR (95% CI)[3]	P-value
Overall	16 / 1,259 (1.1%)	22 / 1,381 (1.2%)	38 / 2,640 (1.3%)	15 / 1,304 (1.0%)	1.18 (0.48 - 2.88)	0.674	1.20 (0.23 - 6.27)	0.794	1.37 (0.43 - 4.37)	0.530	1.02 (0.43 - 2.44)	0.952	1.23 (0.22 - 6.75)	0.775	1.29 (0.39 - 4.3)	0.623
Women	15 / 1,088 (1.3%)	18 / 1,204 (1.1%)	33 / 2,292 (1.4%)	12 / 1,120 (0.9%)	1.44 (0.51 – 4.07)	0.421	1.30 (0.26 - 6.38)	0.702	1.56 (0.50 - 4.83)	0.375	1.23 (0.52 - 2.94)	0.578	1.33 (0.24 - 7.32)	0.692	1.47 (0.46 - 4.67)	0.445
Men[4]	1 / 171 (1.4%)	4 / 177 (2.2%)	5 / 348 (1.4%)	3 / 184 (1.9%)	..	..	..	..	..	..	..	..	..	..	..	..

[1]Prevalence figures are calculated as the geometric means of the prevalence in each community.

[2]PR (95% CI): Prevalence Ratio (95% Confidence Interval).

[3]aPR (95% CI): adjusted Prevalence Ratio (95% Confidence Interval). Overall prevalence ratios were adjusted for age, sex, language(s) used, baseline wealth, baseline position in the HIV care cascade and baseline prevalence for each dimension. Analyses stratified by sex were adjusted for the same variables, except sex was excluded.

[4]The very small number of men reporting problems with self-care means that estimates are not reported for this outcome.

Table A.4. Effect of the universal testing and treatment intervention on problems with daily activities at 36 months.

	Arm A Number reporting / Total (Prevalence[1])	Arm B Number reporting / Total (Prevalence[1])	Arm A + B Number reporting / Total (Prevalence[1])	Arm C Number reporting / Total (Prevalence[1])	Unadjusted prevalence ratios						Adjusted prevalence ratios					
					Arm A vs Arm C		Arm B vs Arm C		Arm A + B vs Arm C		Arm A vs Arm C		Arm B vs Arm C		Arm A + B vs Arm C	
					PR (95% CI)[2]	P-value	PR (95% CI)[2]	P-value	PR (95% CI)[2]	P-value	aPR (95% CI)[3]	P-value	aPR (95% CI)[3]	P-value	aPR (95% CI)[3]	P-value
Overall	27 / 1,259 (1.6%)	55 / 1,381 (3.1%)	82 / 2,640 (2.7%)	30 / 1,304 (2.3%)	0.71 (0.21 - 2.35)	0.505	1.37 (0.45 - 4.21)	0.515	1.20 (0.45 - 3.22)	0.668	1.03 (0.41 - 2.54)	0.947	1.55 (0.44 - 5.50)	0.428	1.55 (0.58 - 4.13)	0.315
Women	24 / 1,088 (1.7%)	44 / 1,204 (2.7%)	68 / 2,292 (2.6%)	24 / 1,120 (1.8%)	0.96 (0.34 - 2.73)	0.935	1.52 (0.43 - 5.33)	0.448	1.43 (0.52 - 3.92)	0.416	1.46 (0.55 - 3.87)	0.377	1.79 (0.39 - 8.13)	0.382	1.94 (0.60 - 6.24)	0.215
Men	3 / 171 (1.9%)	11 / 177 (4.5%)	14 / 348 (3.0%)	6 / 184 (3.4%)	0.55 (0.18 - 1.70)	0.243	1.33 (0.28 - 6.31)	0.688	0.88 (0.20 - 3.86)	0.845	0.71 (0.31 - 1.62)	0.347	1.52 (0.35 - 6.51)	0.510	1.02 (0.27 - 3.78)	0.978

[1]Prevalence figures are calculated as the geometric means of the prevalence in each community.
[2]PR (95% CI): Prevalence Ratio (95% Confidence Interval).
[3]aPR (95% CI): adjusted Prevalence Ratio (95% Confidence Interval). Overall prevalence ratios were adjusted for age, sex, language(s) used, baseline wealth, baseline position in the HIV care cascade and baseline prevalence for each dimension. Analyses stratified by sex were adjusted for the same variables, except sex was excluded.

Table A.5. Effect of the universal testing and treatment intervention on problems with pain/discomfort at 36 months.

	Arm A Number reporting / Total (Prevalence[1])	Arm B Number reporting / Total (Prevalence[1])	Arm A + B Number reporting / Total (Prevalence[1])	Arm C Number reporting / Total (Prevalence[1])	Unadjusted prevalence ratios						Adjusted prevalence ratios					
					Arm A vs Arm C		Arm B vs Arm C		Arm A + B vs Arm C		Arm A vs Arm C		Arm B vs Arm C		Arm A + B vs Arm C	
					PR (95% CI)[2]	P-value	PR (95% CI)[2]	P-value	PR (95% CI)[2]	P-value	aPR (95% CI)[3]	P-value	aPR (95% CI)[3]	P-value	aPR (95% CI)[3]	P-value
Overall	62 / 1,259 (2.7%)	121 / 1,381 (8.2%)	183 / 2,640 (6.6%)	114 / 1,304 (9.0%)	0.30 (0.08 - 1.09)	0.062	0.91 (0.42 - 2.00)	0.786	0.73 (0.39 - 1.35)	0.255	0.37 (0.14 - 0.97)	0.046	1.12 (0.55 - 2.25)	0.717	0.90 (0.58 - 1.39)	0.563
Women	56 / 1,088 (2.8%)	105 / 1,204 (8.2%)	161 / 2,292 (6.6%)	99 / 1,120 (9.0%)	0.31 (0.09 - 1.06)	0.058	0.90 (0.41 - 2.01)	0.767	0.73 (0.40 - 1.33)	0.244	0.38 (0.15 - 0.97)	0.045	1.14 (0.55 - 2.35)	0.684	0.91 (0.59 - 1.42)	0.631
Men	6 / 171 (2.7%)	16 / 177 (7.6%)	22 / 348 (5.4%)	15 / 184 (8.7%)	0.31 (0.10 - 0.93)	0.040	0.87 (0.34 - 2.24)	0.739	0.62 (0.23 – 1.69)	0.286	0.41 (0.20 - 0.83)	0.022	0.94 (0.42 - 2.12)	0.858	0.69 (0.34 - 1.39)	0.243

[1]Prevalence figures are calculated as the geometric means of the prevalence in each community.
[2]PR (95% CI): Prevalence Ratio (95% Confidence Interval).
[3]aPR (95% CI): adjusted Prevalence Ratio (95% Confidence Interval). Overall prevalence ratios were adjusted for age, sex, language(s) used, baseline wealth, baseline position in the HIV care cascade and baseline prevalence for each dimension. Analyses stratified by sex were adjusted for the same variables, except sex was excluded.

Table A.6. Effect of the universal testing and treatment intervention on problems with anxiety/depression at 36 months.

	Arm A Number reporting / Total (Prevalence[1])	Arm B Number reporting / Total (Prevalence[1])	Arm A + B Number reporting / Total (Prevalence[1])	Arm C Number reporting / Total (Prevalence[1])	Unadjusted prevalence ratios						Adjusted prevalence ratios					
					Arm A vs Arm C		Arm B vs Arm C		Arm A + B vs Arm C		Arm A vs Arm C		Arm B vs Arm C		Arm A + B vs Arm C	
					PR (95% CI)[2]	P-value	PR (95% CI)[2]	P-value	PR (95% CI)[2]	P-value	aPR (95% CI)[3]	P-value	aPR (95% CI)[3]	P-value	aPR (95% CI)[3]	P-value
Overall	44 / 1,259 (2.4%)	113 / 1,381 (5.2%)	157 / 2,640 (5.1%)	33 / 1,304 (2.1%)	1.17 (0.22 - 6.31)	0.824	2.49 (0.49 - 12.78)	0.220	2.44 (0.62 - 9.67)	0.163	1.24 (0.28 - 5.51)	0.734	2.56 (0.64 - 10.19)	0.147	2.53 (0.77 - 8.26)	0.103
Women	42 / 1,088 (2.7%)	97 / 1,204 (5.3%)	139 / 2,292 (5.3%)	28 / 1,120 (1.9%)	1.40 (0.23 - 8.44)	0.662	2.72 (0.52 - 14.30)	0.191	2.72 (0.62 - 11.82)	0.147	1.5 (0.32 - 7.03)	0.544	2.88 (0.76 - 10.98)	0.101	2.87 (0.87 - 9.46)	0.073
Men	2 / 171 (1.7%)	16 / 177 (5.3%)	18 / 348 (3.5%)	5 / 184 (2.8%)	0.62 (0.21 - 1.78)	0.306	1.92 (0.41 - 9.11)	0.343	1.28 (0.30 - 5.50)	0.691	0.84 (0.31 - 2.31)	0.688	1.90 (0.36 - 10.01)	0.382	1.34 (0.34 - 5.32)	0.621

[1]Prevalence figures are calculated as the geometric means of the prevalence in each community.
[2]PR (95% CI): Prevalence Ratio (95% Confidence Interval).
[3]aPR (95% CI): adjusted Prevalence Ratio (95% Confidence Interval). Overall prevalence ratios were adjusted for age, sex, language(s) used, baseline wealth, baseline position in the HIV care cascade and baseline prevalence for each dimension. Analyses stratified by sex were adjusted for the same variables, except sex was excluded.

Table A.7. Adjusted effect of the universal testing and treatment intervention on health-related quality of life score at 36 months, when people who seroconverted during the trial were included.

	Arm A vs Arm C		Arm B vs Arm C		Arm A + B vs Arm C	
	aMD (95% CI)[1]	P-value	aMD (95% CI)[1]	P-value	aMD (95% CI)[1]	P-value
Overall	0.001 (-0.003 - 0.006)	0.514	-0.005 (-0.014 - 0.005)	0.260	0 (-0.005 - 0.004)	0.843
Women	0.001 (-0.004 - 0.005)	0.675	-0.005 (-0.014 - 0.004)	0.203	-0.001 (-0.005 - 0.003)	0.603
Men	0.006 (-0.004 - 0.016)	0.173	-0.003 (-0.018 - 0.011)	0.604	0.004 (-0.005 - 0.013)	0.329

[1]aMD (95% CI): adjusted Mean Difference (95% Confidence Interval). Overall mean differences were adjusted for age, sex, language(s) used, baseline wealth, baseline position in the HIV care cascade and baseline health-related quality of life score. Analyses stratified by sex were adjusted for the same variables, except sex was excluded.

Table A.8. Adjusted effect of the universal testing and treatment intervention on problems with dimensions of health-related quality of life at 36 months, when people who seroconverted during the trial were included.

	Arm A vs Arm C		Arm B vs Arm C		Arm A + B vs Arm C	
	aPR (95% CI)[1]	P-value	aPR (95% CI)[1]	P-value	aPR (95% CI)[1]	P-value
Mobility						
Overall	0.67 (0.35 - 1.28)	0.184	1.17 (0.36 - 3.82)	0.753	1.14 (0.54 - 2.45)	0.679
Women	0.73 (0.41 - 1.31)	0.238	1.12 (0.23 - 5.39)	0.860	1.13 (0.38 - 3.41)	0.788
Men	0.68 (0.21 - 2.22)	0.460	1.17 (0.52 - 2.65)	0.654	0.88 (0.39 - 1.99)	0.723
Self-care						
Overall	0.89 (0.40 - 1.96)	0.720	1.09 (0.20 - 5.83)	0.908	1.20 (0.39 - 3.70)	0.705
Women	1.05 (0.46 - 2.40)	0.890	1.14 (0.22 - 5.97)	0.853	1.33 (0.44 - 3.97)	0.553
Men	0.37 (0.12 - 1.11)	0.068	0.86 (0.14 - 5.25)	0.840	0.40 (0.08 - 2.04)	0.217
Daily activities						
Overall	1.08 (0.45 - 2.59)	0.847	1.52 (0.41 - 5.62)	0.460	1.55 (0.57 - 4.22)	0.328
Women	1.53 (0.57 - 4.09)	0.328	1.82 (0.40 - 8.38)	0.372	2.01 (0.62 - 6.58)	0.199
Men	0.61 (0.23 - 1.66)	0.273	1.27 (0.31 - 5.13)	0.689	0.84 (0.24 - 2.96)	0.751
Pain/discomfort						
Overall	0.38 (0.14 - 1.03)	0.055	1.22 (0.62 - 2.41)	0.493	0.96 (0.63 - 1.47)	0.817
Women	0.40 (0.15 - 1.06)	0.061	1.28 (0.65 - 2.51)	0.414	0.99 (0.65 - 1.52)	0.970
Men	0.39 (0.14 - 1.10)	0.067	0.91 (0.38 - 2.16)	0.800	0.66 (0.29 - 1.51)	0.268
Anxiety/depression						
Overall	1.49 (0.37 - 5.99)	0.506	3.07 (0.85 - 11.09)	0.076	2.53 (0.77 - 8.26)	0.103
Women	1.81 (0.42 - 7.74)	0.359	3.48 (1.00 - 12.08)	0.049	2.87 (0.87 - 9.46)	0.073
Men	0.87 (0.33 - 2.31)	0.736	2.13 (0.41 - 10.95)	0.303	1.34 (0.34 - 5.32)	0.621

[1]aPR (95% CI): adjusted Prevalence Ratio (95% Confidence Interval). Overall prevalence ratios were adjusted for age, sex, language(s) used, baseline wealth, baseline position in the HIV care cascade, and baseline prevalence for each dimension. Analyses stratified by sex were adjusted for the same variables, except sex was excluded.

Table A.9. Adjusted effect of the universal testing and treatment intervention on health-related quality of life score at 12 months, when people who seroconverted during the trial were included.

	Arm A vs Arm C		Arm B vs Arm C		Arm A + B vs Arm C	
	aMD (95% CI)[1]	P-value	aMD (95% CI)[1]	P-value	aMD (95% CI)[1]	P-value
Overall	-0.001 (-0.010 - 0.007)	0.769	-0.006 (-0.017 - 0.006)	0.283	-0.002 (-0.012 - 0.007)	0.557
Women	0 (-0.008 - 0.008)	0.951	-0.004 (-0.018 -0.009)	0.471	-0.001 (-0.012 - 0.010)	0.824
Men	-0.010 (-0.023 - 0.003)	0.110	-0.012 (-0.030 -0.007)	0.186	-0.011 (-0.024 - 0.001)	0.067

[1]aMD (95% CI): adjusted Mean Difference (95% Confidence Interval). Overall mean differences were adjusted for age, sex, language(s) used, baseline wealth, baseline position in the HIV care cascade and baseline health-related quality of life score. Analyses stratified by sex were adjusted for the same variables, except sex was excluded.

Table A.10. Adjusted effect of the universal testing and treatment intervention on problems with dimensions of health-related quality of life at 12 months, when people who seroconverted during the trial were included.

	Arm A vs Arm C		Arm B vs Arm C		Arm A + B vs Arm C	
	aPR (95% CI)[1]	P-value	aPR (95% CI)[1]	P-value	aPR (95% CI)[1]	P-value
Mobility						
Overall	0.71 (0.27 - 1.88)	0.422	1.57 (0.66 - 3.75)	0.248	1.28 (0.64 - 2.54)	0.414
Women	0.58 (0.24 - 1.42)	0.189	1.40 (0.64 - 3.06)	0.339	1.09 (0.58 - 2.05)	0.744
Men[2]	..	..	..	..	..	..
Self-care						
Overall	0.92 (0.33 - 2.51)	0.841	1.75 (0.67 - 4.55)	0.201	1.39 (0.57 - 3.41)	0.402
Women	0.92 (0.35 - 2.40)	0.842	1.57 (0.64 - 3.85)	0.267	1.27 (0.52 - 3.08)	0.541
Men[2]	..	..	..	..	..	..
Daily activities						
Overall	0.88 (0.30 - 2.59)	0.790	1.34 (0.51 - 3.51)	0.485	1.23 (0.54 - 2.80)	0.566
Women	0.86 (0.24 - 3.05)	0.785	1.37 (0.46 - 4.08)	0.506	1.22 (0.45 - 3.34)	0.646
Men	1.64 (0.78 - 3.44)	0.152	1.08 (0.30 - 3.95)	0.886	1.23 (0.47 - 3.21)	0.621
Pain/discomfort						
Overall	0.90 (0.39 - 2.08)	0.761	1.54 (0.43 - 5.49)	0.434	1.44 (0.51 - 4.01)	0.423
Women	0.85 (0.36 - 2.01)	0.659	1.54 (0.42 - 5.65)	0.448	1.4 (0.50 - 3.88)	0.451
Men	1.32 (0.63 - 2.77)	0.392	0.82 (0.22 - 3.07)	0.729	1.2 (0.47 - 3.11)	0.649
Anxiety/depression						
Overall	0.70 (0.18 - 2.70)	0.538	1.79 (0.60 - 5.37)	0.242	1.62 (0.63 - 4.17)	0.258
Women	0.63 (0.14 - 2.93)	0.494	1.53 (0.48 - 4.91)	0.403	1.37 (0.48 - 3.86)	0.488
Men	1.85 (0.72 - 4.74)	0.163	1.98 (0.78 - 5.02)	0.121	2.31 (0.91 - 5.87)	0.071

[1]aPR (95% CI): adjusted Prevalence Ratio (95% Confidence Interval). Overall prevalence ratios were adjusted for age, sex, language(s) used, baseline wealth, baseline position in the HIV care cascade, and baseline prevalence for each dimension. Analyses stratified by sex were adjusted for the same variables, except sex was excluded.
[2]The very small number of men reporting problems with self-care and mobility means that estimates are not reported for these outcomes.

Table A.11. Adjusted effect of the universal testing and treatment intervention on health-related quality of life score at 24 months, when people who seroconverted during the trial were included.

	Arm A vs Arm C		Arm B vs Arm C		Arm A + B vs Arm C	
	aMD (95% CI)[1]	P-value	aMD (95% CI)[1]	P-value	aMD (95% CI)[1]	P-value
Overall	-0.002 (-0.007 - 0.004)	0.461	-0.005 (-0.013 - 0.002)	0.144	-0.003 (-0.008 - 0.001)	0.127
Women	-0.001 (-0.005 - 0.003)	0.420	-0.003 (-0.012 - 0.005)	0.374	-0.002 (-0.005 - 0.001)	0.183
Men	0 (-0.010 - 0.011)	0.933	-0.013 (-0.029 - 0.004)	0.120	-0.007 (-0.021 - 0.007)	0.244

[1]aMD (95% CI): adjusted Mean Difference (95% Confidence Interval). Overall mean differences were adjusted for age, sex, language(s) used, baseline wealth, baseline position in the HIV care cascade and baseline health-related quality of life score. Analyses stratified by sex were adjusted for the same variables, except sex was excluded.

Table A.12. Adjusted effect of the universal testing and treatment intervention on problems with dimensions of health-related quality of life at 24 months, when people who seroconverted during the trial were included.

	Arm A vs Arm C		Arm B vs Arm C		Arm A + B vs Arm C	
	aPR (95% CI)[1]	P-value	aPR (95% CI)[1]	P-value	aPR (95% CI)[1]	P-value
Mobility						
Overall	0.79 (0.22 - 2.85)	0.665	1.21 (0.43 - 3.35)	0.670	1.25 (0.52 - 3.01)	0.554
Women	0.84 (0.28 - 2.52)	0.704	0.76 (0.23 - 2.50)	0.596	1.14 (0.53 - 2.44)	0.684
Men	0.71 (0.28 - 1.78)	0.398	1.84 (0.61 - 5.60)	0.227	0.94 (0.29 - 3.05)	0.903
Self-care						
Overall	0.84 (0.36 - 1.96)	0.625	0.99 (0.30 - 3.33)	0.990	1.02 (0.35 - 2.93)	0.967
Women	0.82 (0.43 - 1.59)	0.493	0.78 (0.24 - 2.56)	0.624	0.89 (0.35 - 2.31)	0.782
Men	0.83 (0.25 - 2.72)	0.711	1.75 (0.93 - 3.30)	0.075	0.94 (0.32 - 2.73)	0.888
Daily activities						
Overall	0.73 (0.25 - 2.15)	0.500	1.06 (0.48 - 2.34)	0.873	1.19 (0.80 - 1.75)	0.326
Women	0.79 (0.30 - 2.07)	0.574	0.76 (0.25 - 2.32)	0.570	1.1 (0.74 - 1.63)	0.570
Men	0.67 (0.22 - 2.02)	0.413	1.67 (0.86 - 3.24)	0.109	1.17 (0.56 - 2.46)	0.626
Pain/discomfort						
Overall	0.83 (0.51 - 1.36)	0.398	1.06 (0.53 - 2.14)	0.836	1.04 (0.69 - 1.57)	0.822
Women	0.85 (0.57 - 1.28)	0.374	0.94 (0.45 - 1.97)	0.835	0.99 (0.71 - 1.40)	0.962
Men	0.66 (0.19 - 2.28)	0.441	1.74 (0.56 - 5.39)	0.279	1.29 (0.45 - 3.72)	0.579
Anxiety/depression						
Overall	1.03 (0.34 - 3.12)	0.956	1.64 (0.73 - 3.68)	0.182	1.76 (0.92 - 3.38)	0.079
Women	1.06 (0.36 - 3.11)	0.901	1.62 (0.73 - 3.61)	0.191	1.74 (0.98 - 3.10)	0.056
Men	1.11 (0.25 - 4.92)	0.868	1.41 (0.49 - 4.04)	0.456	1.58 (0.49 - 5.09)	0.378

[1]aPR (95% CI): adjusted Prevalence Ratio (95% Confidence Interval). Overall prevalence ratios were adjusted for age, sex, language(s) used, baseline wealth, baseline position in the HIV care cascade, and baseline prevalence for each dimension. Analyses stratified by sex were adjusted for the same variables, except sex was excluded.

Table A.13. Adjusted effect of the universal testing and treatment intervention on health-related quality of life score at 36 months, among people who were unaware of their status, or aware of their status but not on antiretroviral therapy.

	Arm A vs Arm C		Arm B vs Arm C		Arm A + B vs Arm C	
	aMD (95% CI)[1]	P-value	aMD (95% CI)[1]	P-value	aMD (95% CI)[1]	P-value
Overall	0.004 (-0.001 - 0.009)	0.109	-0.003 (-0.014 - 0.007)	0.452	0.002 (-0.002 - 0.007)	0.290
Women	0.004 (0 - 0.008)	0.036	-0.002 (-0.010 - 0.006)	0.602	0.003 (-0.001 - 0.006)	0.129
Men	-0.003 (-0.021 - 0.015)	0.714	-0.012 (-0.036 - 0.012)	0.259	-0.003 (-0.019 - 0.013)	0.640

[1]aMD (95% CI): adjusted Mean Difference (95% Confidence Interval). Overall mean differences were adjusted for age, sex, language(s) used, baseline wealth, and baseline health-related quality of life score. Analyses stratified by sex were adjusted for the same variables, except sex was excluded.

Table A.14. Adjusted effect of the universal testing and treatment intervention on problems with dimensions of health-related quality of life at 36 months, among people who were unaware of their status, or aware of their status but not on antiretroviral therapy.

	Arm A vs Arm C		Arm B vs Arm C		Arm A + B vs Arm C	
	aPR (95% CI)[1]	P-value	aPR (95% CI)[1]	P-value	aPR (95% CI)[1]	P-value
Mobility						
Overall	0.79 (0.34 - 1.85)	0.519	3.11 (0.64 - 15.01)	0.129	1.48 (0.59 - 3.72)	0.343
Women	0.74 (0.25 - 2.18)	0.520	2.54 (0.45 - 14.29)	0.234	1.25 (0.39 - 4.01)	0.655
Self-care						
Overall	0.82 (0.38 - 1.80)	0.564	1.71 (0.50 - 5.84)	0.329	1.07 (0.37 - 3.10)	0.888
Women	0.80 (0.35 - 1.84)	0.535	1.43 (0.43 - 4.79)	0.500	0.92 (0.30 - 2.79)	0.859
Daily activities						
Overall	0.96 (0.29 - 3.23)	0.940	1.56 (0.29 - 8.38)	0.542	1.39 (0.37 - 5.27)	0.571
Women	1.37 (0.35 - 5.36)	0.590	1.32 (0.29 - 6.09)	0.668	1.25 (0.33 - 4.72)	0.695
Pain/discomfort						
Overall	0.31 (0.08 - 1.16)	0.072	1.08 (0.54 - 2.19)	0.792	0.75 (0.45 - 1.26)	0.224
Women	0.30 (0.09 - 0.96)	0.044	0.89 (0.41 - 1.95)	0.730	0.60 (0.32 - 1.13)	0.097
Anxiety/depression						
Overall	0.69 (0.26 - 1.79)	0.373	1.48 (0.43 - 5.06)	0.468	1.09 (0.53 - 2.24)	0.779
Women	0.77 (0.29 - 2.03)	0.534	1.44 (0.46 - 4.54)	0.462	1.07 (0.54 - 2.14)	0.814

[1]aPR (95% CI): adjusted Prevalence Ratio (95% Confidence Interval). Overall prevalence ratios were adjusted for age, sex, language(s) used, baseline wealth, and baseline prevalence for each dimension. Analyses stratified by sex focused on women only, because the number of men reporting any problems was small. These analyses were adjusted for the same variables, except sex was excluded.

Table A.15. Adjusted effect of the universal testing and treatment intervention on health-related quality of life score at 12 months, among people who were unaware of their status, or aware of their status but not on antiretroviral therapy.

	Arm A vs Arm C		Arm B vs Arm C		Arm A + B vs Arm C	
	aMD (95% CI)[1]	P-value	aMD (95% CI)[1]	P-value	aMD (95% CI)[1]	P-value
Overall	-0.001 (-0.010 - 0.008)	0.761	-0.006 (-0.016 - 0.005)	0.249	-0.003 (-0.014 - 0.008)	0.557
Women	-0.001 (-0.010 - 0.008)	0.805	-0.004 (-0.014 - 0.006)	0.369	-0.002 (-0.013 - 0.009)	0.661
Men	-0.003 (-0.013 - 0.007)	0.543	-0.010 (-0.028 - 0.009)	0.252	-0.005 (-0.019 - 0.008)	0.361

[1]aMD (95% CI): adjusted Mean Difference (95% Confidence Interval). Overall mean differences were adjusted for age, sex, language(s) used, baseline wealth, and baseline health-related quality of life score. Analyses stratified by sex were adjusted for the same variables, except sex was excluded.

Table A.16. Adjusted effect of the universal testing and treatment intervention on problems with dimensions of health-related quality of life at 12 months, among people who were unaware of their status, or aware of their status but not on antiretroviral therapy.

	Arm A vs Arm C		Arm B vs Arm C		Arm A + B vs Arm C	
	aPR (95% CI)[1]	P-value	aPR (95% CI)[1]	P-value	aPR (95% CI)[1]	P-value
Mobility						
Overall	0.85 (0.32 - 2.23)	0.695	1.59 (0.53 - 4.71)	0.339	1.09 (0.38 - 3.09)	0.851
Women	0.60 (0.21 - 1.75)	0.291	1.48 (0.49 - 4.49)	0.421	0.88 (0.30 - 2.62)	0.791
Self-care						
Overall	0.92 (0.27 - 3.13)	0.875	1.52 (0.47 - 4.98)	0.419	1.13 (0.30 - 4.26)	0.833
Women	0.96 (0.30 - 3.01)	0.928	1.21 (0.36 - 4.09)	0.713	0.98 (0.25 - 3.88)	0.974
Daily activities						
Overall	1.44 (0.53 - 3.91)	0.408	2.02 (0.58 - 6.99)	0.216	1.73 (0.60 - 5.00)	0.255
Women	1.25 (0.58 - 2.72)	0.500	1.72 (0.55 - 5.31)	0.287	1.40 (0.53 - 3.69)	0.422
Pain/discomfort						
Overall	0.97 (0.33 - 2.82)	0.940	1.56 (0.50 - 4.92)	0.379	1.33 (0.47 - 3.75)	0.524
Women	0.96 (0.31 - 2.96)	0.934	1.46 (0.47 - 4.52)	0.439	1.22 (0.44 - 3.38)	0.643
Anxiety/depression						
Overall	0.65 (0.17 - 2.51)	0.461	1.56 (0.50 - 4.89)	0.381	1.30 (0.47 - 3.64)	0.550
Women	0.74 (0.17 - 3.22)	0.629	1.39 (0.41 - 4.77)	0.535	1.14 (0.33 - 3.87)	0.805

[1]aPR (95% CI): adjusted Prevalence Ratio (95% Confidence Interval). Overall prevalence ratios were adjusted for age, sex, language(s) used, baseline wealth, and baseline prevalence for each dimension. Analyses stratified by sex focused on women only, because the number of men reporting any problems was small. These analyses were adjusted for the same variables, except sex was excluded.

Table A.17. Adjusted effect of the universal testing and treatment intervention on health-related quality of life score at 24 months, among people who were unaware of their status, or aware of their status but not on antiretroviral therapy.

	Arm A vs Arm C		Arm B vs Arm C		Arm A + B vs Arm C	
	aMD (95% CI)[1]	P-value	aMD (95% CI)[1]	P-value	aMD (95% CI)[1]	P-value
Overall	-0.003 (-0.016 - 0.009)	0.541	-0.005 (-0.013 - 0.003)	0.180	-0.006 (-0.013 - 0.002)	0.113
Women	-0.003 (-0.016 - 0.010)	0.549	-0.002 (-0.012 - 0.007)	0.561	-0.004 (-0.012 - 0.005)	0.284
Men	-0.001 (-0.017 - 0.014)	0.823	-0.009 (-0.018 - 0)	0.058	-0.010 (-0.021 - 0.002)	0.082

[1]aMD (95% CI): adjusted Mean Difference (95% Confidence Interval). Overall mean differences were adjusted for age, sex, language(s) used, baseline wealth, and baseline health-related quality of life score. Analyses stratified by sex were adjusted for the same variables, except sex was excluded.

Table A.18. Adjusted effect of the universal testing and treatment intervention on problems with dimensions of health-related quality of life at 24 months, among people who were unaware of their status, or aware of their status but not on antiretroviral therapy.

	Arm A vs Arm C		Arm B vs Arm C		Arm A + B vs Arm C	
	aPR (95% CI)[1]	P-value	aPR (95% CI)[1]	P-value	aPR (95% CI)[1]	P-value
Mobility						
Overall	1.28 (0.32 - 5.06)	0.681	1.93 (0.55 - 6.82)	0.250	1.69 (0.57 - 5.01)	0.281
Women	0.93 (0.17 - 5.09)	0.924	0.84 (0.12 - 6.04)	0.840	0.95 (0.18 - 5.07)	0.938
Self-care						
Overall	0.90 (0.23 - 3.55)	0.863	1.35 (0.40 - 4.51)	0.567	1.23 (0.31 - 4.90)	0.726
Women	0.95 (0.24 - 3.69)	0.930	1.10 (0.28 - 4.37)	0.866	1.11 (0.27 - 4.52)	0.858
Daily activities						
Overall	0.82 (0.19-3.50)	0.745	1.19 (0.34-4.17)	0.746	1.32 (0.52 - 3.34)	0.487
Women	0.93 (0.28-3.11)	0.886	0.97 (0.27-3.49)	0.952	1.32 (0.60 - 2.92)	0.420
Pain/discomfort						
Overall	0.92 (0.27 - 3.11)	0.874	0.96 (0.33 - 2.81)	0.927	1.07 (0.43 - 2.66)	0.863
Women	1.18 (0.29 - 4.83)	0.779	0.83 (0.21 - 3.33)	0.779	1.20 (0.43 - 3.36)	0.680
Anxiety/depression						
Overall	0.84 (0.16 - 4.41)	0.802	1.12 (0.17 - 7.44)	0.893	1.34 (0.33 - 5.38)	0.625
Women	0.65 (0.15 - 2.75)	0.490	0.90 (0.14 - 5.84)	0.896	1.04 (0.27 - 4.09)	0.944

[1]aPR (95% CI): adjusted Prevalence Ratio (95% Confidence Interval). Overall prevalence ratios were adjusted for age, sex, language(s) used, baseline wealth, and baseline prevalence for each dimension. Analyses stratified by sex focused on women only, because the number of men reporting any problems was small. These analyses were adjusted for the same variables, except sex was excluded.

Table A.19. Adjusted effect of the universal testing and treatment intervention on health-related quality of life score at 36 months, among people who were on antiretroviral therapy.

	Arm A vs Arm C		Arm B vs Arm C		Arm A + B vs Arm C	
	aMD (95% CI)[1]	P-value	aMD (95% CI)[1]	P-value	aMD (95% CI)[1]	P-value
Overall	0.003 (-0.002 - 0.007)	0.168	-0.002 (-0.013 - 0.009)	0.615	0.001 (-0.006 - 0.009)	0.635
Women	0.006 (-0.007 - 0.019)	0.306	0.001 (-0.015 - 0.018)	0.853	0.006 (-0.010 - 0.022)	0.390
Men	0.004 (-0.016 - 0.024)	0.638	-0.009 (-0.022 - 0.004)	0.139	-0.005 (-0.019 - 0.009)	0.435

[1]aMD (95% CI): adjusted Mean Difference (95% Confidence Interval). Overall mean differences were adjusted for age, sex, language(s) used, baseline wealth, and baseline health-related quality of life score. Analyses stratified by sex were adjusted for the same variables, except sex was excluded.

Table A.20. Adjusted effect of the universal testing and treatment intervention on problems with dimensions of health-related quality of life at 36 months, among people who were on antiretroviral therapy.

	Arm A vs Arm C		Arm B vs Arm C		Arm A + B vs Arm C	
	aPR (95% CI)[1]	P-value	aPR (95% CI)[1]	P-value	aPR (95% CI)[1]	P-value
Mobility[2]						
Overall	..	..	..	..	..	..
Women	..	..	..	..	..	..
Self-care[2]						
Overall	..	..	..	..	..	..
Women	..	..	..	..	..	..
Daily activities						
Overall	1.22 (0.48 - 3.10)	0.625	1.47 (0.47 - 4.60)	0.444	1.45 (0.53 - 3.95)	0.395
Women	1.12 (0.40 - 3.12)	0.791	1.2 (0.36 - 4.00)	0.725	1.25 (0.43 - 3.60)	0.625
Pain/discomfort						
Overall	0.74 (0.45 - 1.22)	0.194	1.41 (0.54 - 3.64)	0.415	1.28 (0.66 - 2.47)	0.401
Women	0.64 (0.36 - 1.14)	0.108	1.23 (0.43 - 3.51)	0.649	1.14 (0.56 - 2.33)	0.670
Anxiety/depression						
Overall	1.55 (0.36 - 6.63)	0.486	2.56 (0.52 - 12.53)	0.197	2.58 (0.51 - 13.03)	0.202
Women	1.39 (0.31 - 6.18)	0.609	2.21 (0.44 - 11.02)	0.273	2.24 (0.43 - 11.57)	0.274

[1]aPR (95% CI): adjusted Prevalence Ratio (95% Confidence Interval). Overall prevalence ratios were adjusted for age, sex, language(s) used, baseline wealth, and baseline prevalence for each dimension. Analyses stratified by sex focused on women only, because the number of men reporting any problems was small. These analyses were adjusted for the same variables, except sex was excluded.

[2]Few individuals reported any problems with mobility or self-care, so analyses focused on these outcomes were not performed.

Table A.21. Adjusted effect of the universal testing and treatment intervention on health-related quality of life score at 12 months, among people who were on antiretroviral therapy.

	Arm A vs Arm C		Arm B vs Arm C		Arm A + B vs Arm C	
	aMD (95% CI)[1]	P-value	aMD (95% CI)[1]	P-value	aMD (95% CI)[1]	P-value
Overall	-0.005 (-0.014 - 0.005)	0.261	-0.005 (-0.016 - 0.006)	0.326	-0.004 (-0.014 - 0.006)	0.360
Women	-0.003 (-0.013 - 0.007)	0.475	-0.001 (-0.019 - 0.017)	0.862	-0.001 (-0.014 - 0.011)	0.792
Men	-0.007 (-0.025 - 0.011)	0.371	-0.097 (-0.341 - 0.147)	0.352	-0.011 (-0.038 - 0.016)	0.314

[1]aMD (95% CI): adjusted Mean Difference (95% Confidence Interval). Overall mean differences were adjusted for age, sex, language(s) used, baseline wealth, and baseline health-related quality of life score. Analyses stratified by sex were adjusted for the same variables, except sex was excluded.

Table A.22. Adjusted effect of the universal testing and treatment intervention on problems with dimensions of health-related quality of life at 12 months, among people who were on antiretroviral therapy.

	Arm A vs Arm C		Arm B vs Arm C		Arm A + B vs Arm C	
	aPR (95% CI)[1]	P-value	aPR (95% CI)[1]	P-value	aPR (95% CI)[1]	P-value
Mobility						
Overall	0.92 (0.19 - 4.51)	0.898	1.07 (0.26 - 4.34)	0.912	1.02 (0.22 - 4.79)	0.973
Women	0.81 (0.19 - 3.47)	0.732	0.83 (0.24 - 2.81)	0.717	0.87 (0.22 - 3.48)	0.813
Self-care						
Overall	0.69 (0.17 - 2.80)	0.543	0.91 (0.26 - 3.14)	0.853	0.81 (0.20 - 3.38)	0.736
Women	0.67 (0.20 - 2.31)	0.462	0.67 (0.26 - 1.71)	0.337	0.69 (0.20 - 2.36)	0.490
Daily activities						
Overall	0.51 (0.13 - 2.05)	0.280	0.51 (0.19 - 1.38)	0.148	0.51 (0.15 - 1.69)	0.216
Women	0.60 (0.11 - 3.20)	0.485	0.49 (0.11 - 2.12)	0.280	0.57 (0.13 - 2.47)	0.381
Pain/discomfort						
Overall	1.07 (0.22 - 5.09)	0.919	0.86 (0.17 - 4.34)	0.828	0.94 (0.17 - 5.11)	0.936
Women	0.94 (0.23 - 3.92)	0.924	0.71 (0.13 - 3.89)	0.643	0.85 (0.17 - 4.20)	0.816
Anxiety/depression						
Overall	1.00 (0.24 - 4.11)	0.995	1.12 (0.27 - 4.71)	0.852	1.16 (0.24 - 5.75)	0.823
Women	0.82 (0.17 - 3.92)	0.762	0.80 (0.14 - 4.48)	0.761	1.02 (0.21 - 5.03)	0.976

[1]aPR (95% CI): adjusted Prevalence Ratio (95% Confidence Interval). Overall prevalence ratios were adjusted for age, sex, language(s) used, baseline wealth, and baseline prevalence for each dimension. Analyses stratified by sex focused on women only, because the number of men reporting any problems was small. These analyses were adjusted for the same variables, except sex was excluded.

Table A.23. Adjusted effect of the universal testing and treatment intervention on health-related quality of life score at 24 months, among people who were on antiretroviral therapy.

	Arm A vs Arm C		Arm B vs Arm C		Arm A + B vs Arm C	
	aMD (95% CI)[1]	P-value	aMD (95% CI)[1]	P-value	aMD (95% CI)[1]	P-value
Overall	-0.004 (-0.013 - 0.006)	0.410	-0.005 (-0.016 - 0.006)	0.282	-0.002 (-0.011 - 0.006)	0.475
Women	-0.003 (-0.014 - 0.008)	0.496	-0.003 (-0.015 - 0.008)	0.508	-0.001 (-0.010 - 0.008)	0.773
Men	-0.002 (-0.016 - 0.013)	0.802	-0.015 (-0.041 - 0.011)	0.217	-0.006 (-0.028 - 0.016)	0.517

[1]aMD (95% CI): adjusted Mean Difference (95% Confidence Interval). Overall mean differences were adjusted for age, sex, language(s) used, baseline wealth, and baseline health-related quality of life score. Analyses stratified by sex were adjusted for the same variables, except sex was excluded.

Table A.24. Adjusted effect of the universal testing and treatment intervention on problems with dimensions of health-related quality of life at 24 months, among people who were on antiretroviral therapy.

	Arm A vs Arm C		Arm B vs Arm C		Arm A + B vs Arm C	
	aPR (95% CI)[1]	P-value	aPR (95% CI)[1]	P-value	aPR (95% CI)[1]	P-value
Mobility						
Overall	0.50 (0.19 - 1.30)	0.126	0.77 (0.28 - 2.12)	0.555	0.81 (0.32 - 2.05)	0.591
Women	0.47 (0.19 - 1.13)	0.081	0.77 (0.25 - 2.40)	0.594	0.70 (0.24 - 2.03)	0.445
Self-care						
Overall	1.07 (0.27 - 4.17)	0.910	0.92 (0.17 - 4.90)	0.910	1.05 (0.26 - 4.28)	0.933
Women	0.89 (0.25 - 3.15)	0.822	0.70 (0.13 - 3.69)	0.621	0.95 (0.23 - 3.93)	0.937
Daily activities						
Overall	0.96 (0.56 - 1.64)	0.849	0.87 (0.21 - 3.73)	0.829	1.13 (0.48 - 2.66)	0.740
Women	0.84 (0.43 - 1.64)	0.546	0.76 (0.18 - 3.19)	0.652	0.96 (0.37 - 2.51)	0.918
Pain/discomfort						
Overall	0.97 (0.50 - 1.89)	0.915	1.25 (0.59 - 2.66)	0.499	1.32 (0.69 - 2.52)	0.332
Women	0.89 (0.39 - 2.04)	0.736	1.18 (0.58 - 2.40)	0.597	1.27 (0.69 - 2.32)	0.377
Anxiety/depression						
Overall	1.82 (0.72 - 4.63)	0.168	2.16 (0.53 - 8.75)	0.226	2.36 (0.64 - 8.72)	0.158
Women	2.04 (0.87 - 4.75)	0.086	2.24 (0.57 - 8.86)	0.202	2.54 (0.76 - 8.47)	0.108

[1]aPR (95% CI): adjusted Prevalence Ratio (95% Confidence Interval). Overall prevalence ratios were adjusted for age, sex, language(s) used, baseline wealth, and baseline prevalence for each dimension. Analyses stratified by sex focused on women only, because the number of men reporting any problems was small. These analyses were adjusted for the same variables, except sex was excluded.

4. References

Andrade, L. F., Ludwig, K., Goni, J. M. R., Oppe, M., & de Pouvourville, G. (2020). A French Value Set for the EQ-5D-5L. *PharmacoEconomics*, *38*(4), 413–425. https://doi.org/10.1007/s40273-019-00876-4

Devlin, N. J., & Brooks, R. (2017). EQ-5D and the EuroQol Group: Past, Present and Future. *Applied Health Economics and Health Policy*, *15*. https://doi.org/10.1007/s40258-017-0310-5

Hargreaves, J. R., Krishnaratne, S., Mathema, H., Lilleston, P. S., Sievwright, K., Mandla, N., Mainga, T., Vermaak, R., Piwowar-Manning, E., Schaap, A., Donnell, D., Ayles, H., Hayes, R. J., Hoddinott, G., Bond, V., & Stangl, A. (2018). Individual and community-level risk factors for HIV stigma in 21 Zambian and South African communities: Analysis of data from the HPTN071 (PopART) study. *AIDS*, *32*(6), 783–793. https://doi.org/10.1097/QAD.0000000000001757

Hayes, R. J., Ayles, H., Beyers, N., Sabapathy, K., Floyd, S., Shanaube, K., Bock, P., Griffith, S., Moore, A., Watson-Jones, D., Fraser, C., Vermund, S. H., Fidler, S., Agyei, Y., Baldwin, M., Barnes, M., Bond, V., Burns, D., Chishinga, N., … White, R. (2014). HPTN 071 (PopART): Rationale and design of a cluster-randomised trial of the population impact of an HIV combination prevention intervention including universal testing and treatment - a study protocol for a cluster randomised trial. *Trials*, *15*(1), 1–17. https://doi.org/10.1186/1745-6215-15-57

Hayes, R. J., Donnell, D., Floyd, S., Mandla, N., Bwalya, J., Sabapathy, K., Yang, B., Phiri, M., Schaap, A., Eshleman, S. H., Piwowar-Manning, E., Kosloff, B., James, A., Skalland, T., Wilson, E., Emel, L., Macleod, D., Dunbar, R., Simwinga, M., … Fidler, S. (2019). Effect of Universal Testing and Treatment on HIV Incidence — HPTN 071 (PopART). *New England Journal of Medicine*, *381*(3), 207–218. https://doi.org/10.1056/NEJMoa1814556

Herdman, M., Gudex, C., Lloyd, A., Janssen, M., Kind, P., Parkin, D., Bonsel, G., & Badia, X. (2011). Development and preliminary testing of the new five-level version of EQ-5D (EQ-5D-5L). *Quality of Life Research*, *20*(10), 1727–1736. https://doi.org/10.1007/s11136-011-9903-x

Janssen, M. F., Pickard, A. S., Golicki, D., Gudex, C., Niewada, M., Scalone, L., Swinburn, P., & Busschbach, J. (2013). Measurement properties of the EQ-5D-5L compared to the EQ-5D-3L across eight patient groups: A multi-country study. *Quality of Life Research*, *22*(7), 1717–1727. https://doi.org/10.1007/s11136-012-0322-4

Kastien-Hilka, T., Rosenkranz, B., Sinanovic, E., Bennett, B., & Schwenkglenks, M. (2017). Health-related quality of life in South African patients with pulmonary tuberculosis. *PLoS ONE*, *12*(4), e0174605. https://doi.org/10.1371/journal.pone.0174605

Lartey, S. T., Si, L., de Graaff, B., Magnussen, C. G., Ahmad, H., Campbell, J., Biritwum, R. B., Minicuci, N., Kowal, P., & Palmer, A. J. (2019). Evaluation of the Association Between Health State Utilities and Obesity in Sub-Saharan Africa: Evidence From World Health Organization Study on Global AGEing and Adult Health Wave 2. *Value in Health*, *22*(9), 1042–1049. https://doi.org/10.1016/j.jval.2019.04.1925

Miners, A., Phillips, A., Kreif, N., Rodger, A., Speakman, A., Fisher, M., Anderson, J., Collins, S., Hart, G., Sherr, L., Lampe, F. C., Johnson, M., McDonnell, J., Aderonke, A., Gilson, R., Edwards, S., Haddow, L., Gilson, S., Broussard, C., … Burman, B. (2014). Health-related quality-of-life of people with HIV in the era of combination antiretroviral treatment: A cross-sectional comparison with the general population. *The Lancet HIV*, *1*(1), e32–e40. https://doi.org/10.1016/S2352-3018(14)70018-9

Oppe, M., Devlin, N. J., Van Hout, B., Krabbe, P. F. M., & De Charro, F. (2014). A program of methodological research to arrive at the new international eq-5d-5l valuation protocol. *Value in Health*, *17*(4), 445–453. https://doi.org/10.1016/j.jval.2014.04.002

Pickard, A. S., Law, E. H., Jiang, R., Pullenayegum, E., Shaw, J. W., Xie, F., Oppe, M., Boye, K. S., Chapman, R. H., Gong, C. L., Balch, A., & Busschbach, J. J. V. (2019). United States Valuation of EQ-5D-5L Health States Using an International Protocol. *Value in Health*, *22*(8), 931–941. https://doi.org/10.1016/j.jval.2019.02.009

Rabin, R., Gudex, C., Selai, C., & Herdman, M. (2014). From translation to version management: A history and review of methods for the cultural adaptation of the euroqol five-dimensional questionnaire. *Value in Health*,

17(1), 70–76. https://doi.org/10.1016/j.jval.2013.10.006

Thomas, R., Burger, R., Harper, A., Kanema, S., Mwenge, L., Vanqa, N., Bell-Mandla, N., Smith, P. C., Floyd, S., Bock, P., Ayles, H., Beyers, N., Donnell, D., Fidler, S., Hayes, R., Hauck, K., Hargreaves, J., Watson-Jones, D., Godfrey-Faussett, P., … Hughes, E. (2017). Differences in health-related quality of life between HIV-positive and HIV-negative people in Zambia and South Africa: a cross-sectional baseline survey of the HPTN 071 (PopART) trial. *The Lancet Global Health*, *5*(11), e1133–e1141. https://doi.org/10.1016/S2214-109X(17)30367-4

Tran, B. X., Ohinmaa, A., & Nguyen, L. T. (2012). Quality of life profile and psychometric properties of the EQ-5D-5L in HIV/AIDS patients. *Health and Quality of Life Outcomes*, *10*(1), 132. https://doi.org/10.1186/1477-7525-10-132

Van Hout, B., Janssen, M. F., Feng, Y. S., Kohlmann, T., Busschbach, J., Golicki, D., Lloyd, A., Scalone, L., Kind, P., & Pickard, A. S. (2012). Interim scoring for the EQ-5D-5L: Mapping the EQ-5D-5L to EQ-5D-3L value sets. *Value in Health*, *15*(5), 708–715. https://doi.org/10.1016/j.jval.2012.02.008

Vermund, S. H., Fidler, S. J., Ayles, H., Beyers, N., & Hayes, R. J. (2013). Can combination prevention strategies reduce HIV transmission in generalized epidemic settings in Africa? The HPTN 071 (PopART) study plan in South Africa and Zambia. *Journal of Acquired Immune Deficiency Syndromes*, *63*(SUPPL. 2), 221–227. https://doi.org/10.1097/QAI.0b013e318299c3f4

Welie, A. G., Gebretekle, G. B., Stolk, E., Mukuria, C., Krahn, M. D., Enquoselassie, F., & Fenta, T. G. (2020). Valuing Health State: An EQ-5D-5L Value Set for Ethiopians. *Value in Health Regional Issues*, *22*, 7–14. https://doi.org/10.1016/j.vhri.2019.08.475

Wu, A. W., Hanson, K. A., Harding, G., Haider, S., Tawadrous, M., Khachatryan, A., Pashos, C. L., & Simpson, K. N. (2013). Responsiveness of the MOS-HIV and EQ-5D in HIV-infected adults receiving antiretroviral therapies. *Health and Quality of Life Outcomes*, *11*, 42. https://doi.org/10.1186/1477-7525-11-42

Yang, F., Katumba, K. R., Roudijk, B., Yang, Z., Revill, P., Griffin, S., Ochanda, P. N., Lamorde, M., Greco, G., Seeley, J., & Sculpher, M. (2022). Developing the EQ-5D-5L Value Set for Uganda Using the ‘Lite’ Protocol. *PharmacoEconomics*, *40*(3), 309–321. https://doi.org/10.1007/s40273-021-01101-x

5. HPTN 071 (PopART) data sharing policy

HPTN 071 (PopART) Data Sharing policy

Background

NIAID, HPTN, LSHTM, and other PopART partners have general expectations and/or policies to make research data available to other researchers in a collaborative manner. However, there is need for an agreed, comprehensive and detailed data sharing policy specific to this study to be widely shared so that all stakeholders share the same expectations for when and what data will be made available, and through what mechanisms. Such a policy will ensure that HPTN 071 protocol team members and stakeholders have a period of priority access to the data, while still providing a pathway for more public access over time, and will strive to ensure that public use represents the data responsibly. As well, a comprehensive policy will avoid reliance on multiple "one-off" data sharing policies or mechanisms that might otherwise arise and be inconsistent with one another. It will enable the team to address expectations and requirements of funding agencies, journals, conferences and the broader scientific community, and will maximise the public policy and health benefit that may be derived from the data.

Types of HPTN 071 (PopART) Data Addressed by this Policy

There are two main types of HPTN071 study data:

1. Data obtained from the population cohort (PC)
    a. Survey data collected by PC field teams
    b. Data obtained from analysis of blood samples obtained from PC participants during household visits by the PC field teams
2. Data obtained by intervention field teams
    a. Data obtained during household visits in Arm A & B communities. Intervention data available aggregated by sex, age group and community.

Principles forming the basis of the HPTN 071 (PopART) data sharing policy

- The HPTN 071 (PopART) protocol team agrees that wider access to research data is in the public interest.

- Secondary analyses arising as a result of data sharing should meet the same high standards expected of the HPTN and HPTN 071 (PopART) analyses.

- The HPTN is committed to building scientific capacity among early career investigators at the research sites in Africa, and this requires that the study team should have exclusive access to the data for a defined period.

- Data arising from research with human subjects have special ethical considerations in terms of confidentiality and consent.
- Because the HPTN 071 (PopART) study is an interventional trial designed to answer a specific set of research questions, the data may not always be appropriate to answer other questions.

- Conclusions derived from misunderstandings or erroneous analyses of data can harm the reputation of a study, destroy the trust of the community, and discourage further participation in research. Therefore, the HPTN 071 (PopART) protocol team has a responsibility to ensure that data are accessed only by legitimate investigators who have agreed to abide by the provisions of this policy.

Key aspects of the HPTN 071 (PopART) data sharing policy

- Researchers from the HPTN 071 (PopART) study team who collected data have a legitimate interest in benefiting from their investment of time and effort, as well as a commitment to supporting capacity building for early career investigators at the study sites. Therefore, the study team will have a period of *exclusive use* before the data are made available for sharing.

    1. Exclusive use will be for a fixed period of 1 year after the publication of the primary results in the New England Journal of Medicine on 18 July 2019.
    2. De-identified analysis datasets for the primary publication will be released to 3ie following publication of the primary manuscript, for replication purposes. Analysis datasets supporting other manuscripts will be posted as required by journals at the time of publication.
    3. Data may be made available to researchers external to the study team earlier, with specific permission of the Protocol Chairs and Protocol Statistician when this does not conflict with the publication plans for the study.
- During and after the period of exclusive protocol team use, protocol team members with approved publication concepts are provided access to HPTN 071 PopART data stored at the

HPTN SDMC by submission of a signed HPTN 071 Data Access Agreement. Protocol team members agree that they will only use the data for the analyses in the approved publication concept.

- During and after the period of exclusive protocol team use, protocol team members may use the data to pursue analyses agreed upon by protocol team leadership but not explicitly described in an approved publication concept, especially those requested by study funders or policymakers.

- After the period of exclusive use, data will be made available to users outside of the protocol team after an application and approval process (controlled public access).

- Requests for data are made in writing to the HPTN 071 (PopART) Publications Working Group, using a standard Data Access Proposal Form. Proposals are reviewed by the Publications Working Group, and access to study data is facilitated by the HPTN Statistical and Data Management Center (SDMC).

- Researchers external to the study protocol team who are granted access to data are encouraged to engage with the HPTN 071 (PopART) study team to ensure they have sufficient understanding of the study and the data elements.

- Any publications arising from the shared data must acknowledge the investigators who collected the data, the institutions involved, and the funding sources. A standard acknowledgement statement will be provided.

Storage for data sharing

- Data will be stored securely at the HPTN SDMC according to HPTN and HPTN 071 (PopART) Standard Operating Procedures (SOPs) and access provided to approved applicants.

- Authorized users will be granted access to all study data relevant to addressing the research question posed.

Standards for data sharing

Data cannot be used effectively unless they are thoroughly documented and their collection methods

understood. Therefore, in general, data are made available for sharing when they are cleaned, documented and the appropriate metadata are in place. Documentation includes detailed descriptions of how the data were collected and coded, and how lab results were obtained. The annotated questionnaire will also be included. Data available for sharing will be from the final 'locked' database.

The following standards will be observed:

- Data will be de-identified before release for sharing, with all direct personal identifiers removed. Where there are indirect identifiers that could lead to deductive disclosure (e.g. GPS coordinates for a person's house), these will be modified or removed from the dataset. Any requests for access to a dataset that includes identifiers will need to be negotiated with the HPTN 071 (PopART) Publications Working Group. If necessary, such requests may need to be referred to relevant research and ethics committees.

- Data may be in separate files, but each file will have a data dictionary that identifies the key fields needed to merge files

- Data documentation will also include the names and institutions of the PIs who collected the data and details of the funding source of the study

Conditions of access for non-protocol team members

All requests to share data must be made in writing using the HPTN 071 (PopART) Data Access Application Form. Where relevant, an accompanying proposal detailing the study questions and analysis methods should be submitted as an appendix to the application form. Applications will be reviewed by the HPTN 071 Publications Working Group.

In order for access to be considered, the applicant must meet the following requirements:

- provide evidence of being a bona fide researcher in a field relevant to the study or on associated statistical methods (e.g. relevant peer-reviewed research that can be found on PubMed, or a successful application to a relevant, independent data access committee). If the primary investigator who will be using the data does not meet these criteria, they must identify a mentor who will take responsibility for supervising them and guaranteeing their compliance with this policy
- have an experienced statistician on their research team
- be willing to sign a data access agreement

- ☐ agree to notify the HPTN SDMC if any errors are identified in the data
- ☐ agree not to share the data with third parties without permission from the HPTN 071 Publications Working Group
- ☐ agree to share the methods and results of all the analyses using HPTN 071 (PopART) data with the HPTN 071 protocol chairs and protocol statistician when completed, and certainly no later than when analyses have been accepted for publication or presentation.

Priority will be given to applicants whose proposals include a plan for sharing analysis skills with site researchers. Access decisions will take into consideration the planned publications by the HPTN 071 (PopART) protocol team. The committee will make its decision within 4 weeks of the application being received. The aim will be that the data will then be transferred within 4 weeks after a positive decision. Where this is not possible, the Protocol Statistician at the HPTN SDMC will inform the HPTN 071 (PopART) Publications Working Group and the applicant in writing and will negotiate a later date.

Any publications arising from the shared data must acknowledge the research team who collected the data, the institutions involved, and funding sources, and must state that HPTN 071 (PopART) investigators were not involved in the analysis (except in the case where there are protocol team collaborators). This acknowledgement statement will be provided to investigators with whom HPTN 071 (PopART) data are shared.
